# Cellular Immune Responses to Live Attenuated Japanese Encephalitis (JE) Vaccine SA14-14-2 in Adults in a JE/Dengue Co-Endemic Area

**DOI:** 10.1371/journal.pntd.0005263

**Published:** 2017-01-30

**Authors:** Lance Turtle, Filippo Tatullo, Tanushka Bali, Vasanthapuram Ravi, Mohammed Soni, Sajesh Chan, Savita Chib, Manjunatha M. Venkataswamy, Prachi Fadnis, Mansour Yaïch, Stefan Fernandez, Paul Klenerman, Vijaya Satchidanandam, Tom Solomon

**Affiliations:** 1 Institute of Infection and Global Health, University of Liverpool, Liverpool, United Kingdom; 2 NIHR Health Protection Research Unit for Emerging and Zoonotic Infections, University of Liverpool, Liverpool, United Kingdom; 3 Tropical & Infectious Disease Unit, Royal Liverpool University Hospital, Liverpool, United Kingdom; 4 Dept of Neurovirology, National Institute of Mental Health and Neurosciences (NIMHANS), Bengaluru, India; 5 Dept of Microbiology and Cell Biology, Indian Institute of Science, Bengaluru, India; 6 Vaxyn Consulting, Paris, Île-de-France, France; 7 Dept of Virology, Armed Forces Research Institute of Medical Science (AFRIMS), Bangkok, Thailand; 8 Translational Gastroenterology Unit, Nuffield Dept. of Medicine, University of Oxford, Peter Medawar Building for Pathogen Research, Oxford, United Kingdom; 9 Walton Centre NHS Foundation Trust, Liverpool, United Kingdom; CDC, UNITED STATES

## Abstract

**Background:**

Japanese encephalitis (JE) virus (JEV) causes severe epidemic encephalitis across Asia, for which the live attenuated vaccine SA14-14-2 is being used increasingly. JEV is a flavivirus, and is closely related to dengue virus (DENV), which is co-endemic in many parts of Asia, with clinically relevant interactions. There is no information on the human T cell response to SA14-14-2, or whether responses to SA14-14-2 cross-react with DENV. We used live attenuated JE vaccine SA14-14-2 as a model for studying T cell responses to JEV infection in adults, and to determine whether these T cell responses are cross-reactive with DENV, and other flaviviruses.

**Methods:**

We conducted a single arm, open label clinical trial (registration: clinicaltrials.gov NCT01656200) to study T cell responses to SA14-14-2 in adults in South India, an area endemic for JE and dengue.

**Results:**

Ten out of 16 (62.5%) participants seroconverted to JEV SA14-14-2, and geometric mean neutralising antibody (NAb) titre was 18.5. Proliferation responses were commonly present before vaccination in the absence of NAb, indicating a likely high degree of previous flavivirus exposure. Thirteen of 15 (87%) participants made T cell interferon-gamma (IFNγ) responses against JEV proteins. In four subjects tested, at least some T cell epitopes mapped cross-reacted with DENV and other flaviviruses.

**Conclusions:**

JEV SA14-14-2 was more immunogenic for T cell IFNγ than for NAb in adults in this JE/DENV co-endemic area. The proliferation positive, NAb negative combination may represent a new marker of long term immunity/exposure to JE. T cell responses can cross-react between JE vaccine and DENV in a co-endemic area, illustrating a need for greater knowledge on such responses to inform the development of next-generation vaccines effective against both diseases.

**Trial Registration:**

clinicaltrials.gov (NCT01656200)

## Author Summary

The *Flavivirus* genus member Japanese encephalitis (JE) virus (JEV), causes severe brain disease in tens of thousands of children across Asia every year. JE is vaccine preventable, and the immune response to JEV plays a major role in disease outcome. However, the response to JEV is hard to study as JE affects young children in rural areas. Related flaviviruses, such as dengue virus (which has no good vaccine), can influence the outcome of JE, probably due to cross-reactive immune responses. T cells (a subset of white blood cells) respond to virus infections, but we know little about the timing and nature of T cell responses to JEV after infection and whether T cells are protective against JEV.

We used the live JE vaccine SA14-14-2 as a model to study the immune response to JEV. We found T cell responses frequently after JE vaccination. In this small group of volunteers, many of whom were exposed to dengue virus, most of the T cell responses tested cross-reacted between JEV and dengue virus. However, only about two thirds of people made antibody responses to the vaccine. Studying these responses could help design new vaccines for use against JE and dengue in Asia.

## Introduction

Japanese encephalitis (JE) virus (JEV) is the cause of around 68 000 cases of encephalitis per year in Asia, mostly in children [1]. JEV is a single stranded positive sense RNA virus of the family *Flaviviridae*, genus *Flavivirus*. The JEV genome is 11 kb comprising a single 10.3 kb open reading frame encoding three structural proteins (core, C; pre-membrane, prM; envelope, E) and seven non-structural (NS) proteins denoted NS1, NS2a, NS2b, NS3, NS4a, NS4b and NS5 [2]. JEV is transmitted naturally among birds and pigs by *Culex* mosquitoes, with humans infected coincidentally as dead-end hosts. Ecological control of JE is, therefore, unrealistic: vaccination is the only reasonable prospect of preventing disease in humans [3].

JE vaccines are effective, have been available for many decades [4], and appear to protect through neutralising antibody (NAb) against JEV [5]. Early JE vaccines were inactivated; subsequently an infectious attenuated vaccine (JEV SA14-14-2) has been developed which is safe and immunogenic [6–8]. In JE endemic areas a single dose is 94.5% to 99.3% effective [9, 10] and gives durable protection for up to five years [11, 12]. The vaccine was prequalified by the World Health Organisation in October 2013 [13].

JEV co-circulates in many parts of Asia with the related flavivirus dengue virus (DENV), currently the target of several developmental vaccines. T cell and antibody responses to DENV are cross-reactive, with clinically relevant effects, both potentially beneficial and harmful [14]. The sequence of exposure to JEV and DENV may also be relevant; DENV partially protects against JE [15], whereas JEV may predispose to worse dengue disease [16]. Cross-reactivity between DENV and other flaviviruses is less well studied, though we have recently described highly cross-reactive CD8^+^ T cell responses between JEV and DENV in South India, associated with asymptomatic exposure to JEV [17].

In addition to their clinical use, live attenuated vaccines may serve as models for viral infection in humans and allow the study of the development of anti-viral immune responses [18, 19]. Greater knowledge of cellular responses to both JEV and JE vaccine was identified as research priority in a JE vaccine Cochrane review [20]. A protective role for T cell responses against JE is not clearly established, but both animal and human studies suggest a role for the cellular response as well as NAb in protection and/or recovery from JE [17, 21–23].

In JE endemic areas most of the population are exposed by adulthood [24]. Therefore, live JE vaccination may mimic repeated exposure to wild type JEV in an immune host, giving information on the T cell response to wild type JEV as well as the vaccine. Although the live JE vaccine is predominantly used in children, the repeated blood sampling required makes such studies impractical in this age group. For these reasons we conducted an exploratory study of T cell responses after vaccination of adults with a single dose of JE vaccine SA14-14-2 in South India, a JE endemic area. Because dengue and JE vaccines will ultimately be used together in much of Asia, and South India is also dengue endemic, we also sought to determine whether T cell responses to JE vaccine could cross-react with DENV (or other flaviviruses), and whether there were JEV-specific T cell responses. Here, we report the first description of T cell responses to live attenuated JE vaccine SA14-14-2 in humans.

## Methods

### Participants

Healthy adults aged 18 to 50 years were recruited into the study by advertisement and word of mouth and vaccinated at the Indian Institute of Science (IISc) or National Institute of Mental Health and Neurosciences (NIMHANS), both in Bengaluru, Karnataka State, India. Any laboratory workers who were being vaccinated because of potential occupational exposure to JEV were eligible. Because of concern that recruitment would be insufficient, an interventional protocol was developed to enrol additional participants. Participants who were being vaccinated on this protocol were screened for anti-JEV NAb before trial entry as JE vaccination was deemed more readily justified if NAb was not detectable. Participants with positive NAb or ELISpot screening assays were included in an observational study [17]. Participants on the interventional protocol also had HIV, hepatitis B and C excluded before entry. Apart from the pre-vaccination screening, both sets of participants followed an identical protocol and were analysed as one group. Exclusion criteria were previous administration of JE vaccine, pregnancy, immunosuppression of any cause, allergy or adverse reaction to a vaccine or component of the investigational vaccine, previous episode of encephalitis, use of any other investigational drug or vaccine within 30 days of vaccination.

### Study design, sample size, endpoints and analysis

This was an open label single arm study. The target sample size was 20, chosen to give a reasonable chance of representing common HLA types in the South Indian population. No power calculation was performed and no comparative analysis was pre-specified. The primary endpoint was a description of the timing, magnitude, specificity and cross-reactivity of the T cell response to JE vaccine SA14-14-2 up to 8 weeks after vaccination. The number of participants seroconverting to the vaccine (defined as NAb titre > 1:10 if negative pre-vaccine, or a four-fold increase over baseline titre), geometric mean NAb titre, the number of adverse events occurring one month after vaccination and number of serious adverse events at any time were secondary endpoints. Data were analysed descriptively; statistics were performed using R version 3.1.2 (www.r-project.org).

### Study procedures

Pre-vaccine samples were collected before subcutaneous injection of 0.5 ml attenuated JE vaccine SA14-14-2 (Chengdu Biological Products, China) over the deltoid by a study physician (lot numbers 201107C017-1, 201107C021-2, 201103C002-2 or 201206C030-2; derived from primary hamster kidney cells). Blood (40-50ml) was drawn at 1, 2, 4 and 8 weeks and monthly thereafter (in some participants). Peripheral blood mononuclear cells (PBMC) and serum were separated and cryopreserved.

### Safety

Safety was assessed actively using weekly symptom diaries for the first four weeks and passively thereafter. Participants were asked about any symptoms at each contact up to six months and were telephoned at this point if face-to-face contact was not possible. Adverse events were graded 1 (symptoms but no change in behaviour), 2 (symptoms sufficient to interfere with usual daily activities), 3 (symptoms prompting medical consultation) or 4 (hospital admission).

### Ethics & consent

The study was conducted according to the principles of the Declaration of Helsinki. All participants gave written, informed consent separately for screening and then for administration of the vaccine. The protocol was approved by the IISc Institutional Human Ethics Committee (ref 5/2011). The observational study was also approved by the Liverpool school of Tropical Medicine ethics committee (ref. 10.59). The interventional protocol was registered at clinicaltrials.gov (NCT01656200).

### Peptides and antigens

A library of 18 amino acid peptides overlapping by 10 corresponding to the entire JEV SA14-14-2 open reading frame based on the two sequences available in Genbank in 2010, accession numbers AF315119 and D90195 (see S1 JEV peptide library), was synthesised commercially (Mimotopes). Peptides were dissolved in dimethylsulphoxide (DMSO) and pooled according to JEV proteins: C/prM, E (2 pools), NS1, NS2a/NS2b, NS3 (2 pools), NS4a/NS4b, NS5 (3 pools). For proliferation assays adjacent pools (except C/prM) were combined. In cross-reactivity assays, the following peptide sets, obtained through Biodefense and Emerging Infection (BEI) Resources, NIAID, NIH, were used: DENV1 Singapore/S275/1990 E (NR4551), NS1 (NR2751), NS3 (NR2752); DENV2 New Guinea C (NGC) prM (NR506), E (NR507), NS1 (NR508), NS3 (NR509); DENV3 Philippines/H87/1956 NS1 (NR2753), NS3 (NR2754); DENV4 Dominica/814669/1981 E (NR512), DENV4 Singapore/8976/1995 NS1 (NR2755), NS3 (NR2756); West Nile virus NY99-flamingo382-99 prM (NR433), M (NR434), E (NR435), NS1 (NR436), NS3 (NR439). JEV infected cell lysate was prepared from Vero cells which were infected with JEV P20778 (MOI 5), fixed with 0.025% glutaraldehyde (Sigma), washed with phosphate buffered saline, suspended in MEM/10% FCS and sonified in a Branson cup-horn sonifier (Model 450, Branson Ultrasonics, Danbury, CT) as previously described [25]. The antigen preparation was diluted to a stock containing 4 μg/ml of JEV E protein and used at a final concentration of 80 ng/ml.

### Interferon-gamma enzyme linked immunospot assay

Interferon-gamma (IFNγ) enzyme linked immunospot (ELISpot) assays were conducted as previously described [17], using 2 x 10^5^ fresh PBMC in triplicate, with peptides at 3 μg/ml and a final DMSO concentration of 0.5% (pools), or 3 μg/ml and DMSO <0.001% (individual peptides). PBMC were cultured in 100 μl RPMI supplemented with 2mM L-glutamine, 100U/ml penicillin, 0.1mg/ml streptomycin (sRPMI) and 10% fetal calf serum (FCS, R10). The cut-off for a positive ELISpot was at least 50 spot forming cells (SFC)/10^6^ PBMC and twice the background count.

### Proliferation assay

Proliferation assays used cryopreserved PBMC which were thawed, then rested overnight before labelling with Carboxyfluorescein succinimidyl ester (CFSE) as previously described [17]. Briefly, PBMC were labelled at 5–10 x 10^6^ cells/ml of pre-warmed phosphate buffered saline (PBS)/1μM CFSE at 37°C for 10 minutes, followed by quenching with five volumes of ice cold R10 and two washes. After eight days in culture with 3 μg/ml JEV peptide pools, cells were stained with near-infra-red (IR) viability dye (molecular probes), anti-CD3-AF700 (clone UCHT1), anti-CD4-PE (clone RPA-T4), anti-CD8-APC (clone RPA-T8) and anti-CD38-PE-Cy7 (clone HIT2) fluorescent antibodies (all from BD biosciences) for flow cytometry.

### Epitope mapping and expansion of short term T cell lines

Peptide epitope mapping was done either by ELISpot, using an additional blood sample if the volunteer was available, or by expanding short term T cell lines (TCL) using left over PBMC. For cross-reactivity assays, short-term T cell lines (TCL) were always used for reasons of consistency. PBMC (2 x 10^6^) were cultured with 5 μg/ml JEV peptide pools, or 10 μg/ml individual peptide, to which responses had been detected in ELISpot assays, in 1 ml sRPMI supplemented with 10% human serum, 10% Natural T cell growth factor/IL2 (“T-stim,” Helvetica Healthcare) and 20 ng/ml recombinant human IL7 (R&D systems). TCLs were expanded for 7–10 days in culture, rested overnight in R10 without peptide, then stimulated with peptides for six hours in the presence of 10 μg/ml brefeldin A (Sigma).

### Intracellular cytokine staining

Intracellular cytokine staining (ICS) assays were done using whole blood, TCL (6 hours) or PBMC (overnight), stimulated with JEV peptides (3–10 μg/ml), peptide pools (3 μg/ml), JEV infected cell lysate, or approximately 10^3.4^ to 10^4.4^ plaque forming units (PFU) of JEV SA14-14-2 in the presence of 10 μg/ml brefeldin A during the stimulation. Following stimulation (and red cell lysis in the case of assays using whole blood), cells were stained with near infrared viability dye (Invitrogen) at room temperature in the dark for 20 minutes, fixed with 2% formaldehyde at room temperature for 20 minutes, and cryopreserved at -80°C in PBS/1% bovine serum albumin/10% DMSO. Later, cells were incubated in FACS perm/wash buffer (BD) at room temperature for 20–30 minutes followed by staining in perm/wash buffer for 30 minutes at 4°C. Antibody clones used for anti-CD3, CD4 and CD8 were as above. Antibodies used for TCL ICS were: anti-CD3-FITC, anti-CD4-PerCP-Cy5.5, anti CD8-APC, anti-IFNγ-PE or PE-Cy7 (clone 4S.B3), anti-IL2-PE (clone 5344.111) and anti-TNFα-PE-Cy7 (clone MAb11). Antibodies used for *ex-vivo* ICS were: anti-CD3-AmCyan, anti-CD4-PerCP-Cy5.5, anti-CD8-Horizon v450, Anti-CD14-APC-Cy7 (clone MφP9), anti-IFNγ-PE-Cy7, anti-TNFα-APC, anti-IL2-PE and anti-MIP-1β-FITC (clone D21-1351). MIP-1β was from R&D systems, all other antibodies were from BD.

### Flow cytometry

Flow cytometry was performed using a BD Canto (TCL ICS and proliferation assays) or Canto II (*ex-vivo* ICS) cytometer. *Ex-vivo* ICS responses were considered positive if the responding population was at least 0.02% of the parent gate and double the negative control value. Proliferation responses were considered positive if the CFSE^lo^/CD38^hi^ responding subset were at least 1% of the parent gate and double the negative control value. Analysis of T cells stained for >2 cytokines was done using Simplified Presentation of Incredibly Complex Evaluations (SPICE) software version 5.35 with pre-processing in Pestle version 1.7 [26].

### Virus neutralisation assays

Screening assays before vaccination measured the ability of heat inactivated sera at two fold dilutions from 1:4 to 1:32 to prevent destruction of a monolayer of PS cells infected with 100 plaque forming units of JEV P20778. Fifty percent plaque reduction neutralisation titres (PRNT_50_) were measured using LLC-MK_2_ cells for all study samples together at the end of the study using the same batch of cells and JEV stock to minimize variation in the assay. For PRNT_50_, sera were heat inactivated and assayed according to the method of Russell *et al* [27]. Viruses used were JEV SA14-14-2 (expanded by three passages in C6/36 cells), DENV1 16007, DENV2 16681, DENV3 16562, DENV4 C0036/06. PRNT_50_ values were calculated by probit regression.

## Results

### Participants

Seventeen participants were recruited into the study (Table 1); nine participants were vaccinated for occupational reasons and seven were vaccinated on the interventional protocol. Median age was 25 years, range 20–39 years. One participant withdrew after a week, a second donated 5ml per sample so had limited assays performed; both remained in the study for safety. Therefore 17 participants were evaluated for safety, 16 for seroconversion, 15 for T cell immunogenicity over 8 weeks, and nine for immunogenicity beyond 8 weeks. Six adverse events in three participants were reported in total, in the first 4 weeks two grade 1 and two grade 2 adverse events occurred, two further grade 2 events occurred >4 weeks after vaccination (Table 1). Four were febrile illnesses (two within 4 weeks), the others were dizziness with, and without, headache. All adverse events recovered spontaneously and no serious adverse events occurred.

**Table 1:** Characteristics of the participants and adverse reactions to live JE vaccine SA14-14-2.

Study participant	Sex	Age at vaccination	Date of vaccination	Time of final sample	Any adverse event (AE)	Number of AEs	Date of AE	Trial Week	Nature of AE	Grade	Duration
VA001/1	Female	28	31/07/2012	16 weeks	No	0					
VA004/1	Male	25	27/07/2012	16 weeks	No	0					
VA007/1	Male	23	27/07/2012	16 weeks	No	0					
VA017/1	Male	25	17/10/2012	26 weeks	No	0					
VA018/1	Male	25	02/11/2012	26 weeks	No	0					
VA020/1	Male	22	19/10/2012	26 weeks	Yes	2	13/11/2012	4	Fever, headache, myalgia, sore throat	2	6 days
							27/11/2012	6	Fever, dry cough	2	7 days
VA023/1	Male	22	23/03/2013	8 weeks	No	0					
VA005/3	Male	29	17/10/2012	26 weeks	No	0					
VA008/3	Female	29	30/05/2012	8 weeks	Yes	2	30/05/2012	1	Dizziness, Headache	1	2–3 hours
							17/06/2012	3	Fever	2	2 days
VA010/3	Female	29	30/05/2012	4 weeks	Yes	2	15/06/2012	3	Dizziness for 2–3 seconds a time	1	10 days
							17/08/2012	12	Fever, myalgia	2	7 days
VA012/3	Male	20	22/10/2012	26 weeks	No	0					
VA015/3	Male	39	22/10/2012	26 weeks	No	0					
VA017/3	Female	38	22/10/2012	1 week*	No	0					
VA019/3	Male	26	23/03/2013	8 weeks	No	0					
VA020/3	Male	27	22/03/2013	8 weeks	No	0					
VA022/3	Male	22	09/04/2013	8 weeks	No	0					
VA023/3	Male	24	11/04/2013	8 weeks	No	0					

Characteristics of the 17 participants and reactions to the vaccine in the study are shown. Adverse events (AE) occurred in three participants, the other 14 participants did not report any adverse events.

*This participant withdrew after one week.

### Generation of antibody and cellular responses

**Fig 1:**
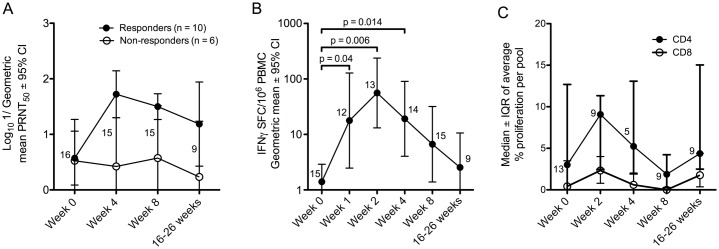
Antibody and cellular responses to JE vaccine SA14-14-2. (A) Neutralisation of JEV SA14-14-2 by sera from vaccinated participants at 4 weeks, 8 weeks and 4–6 months after vaccination. Data are the logarithm of the geometric mean of 1/plaque reduction neutralisation titre 50% (PRNT_50_) ± 95% confidence interval (CI). Filled circles = responders, open circles = non-responders. The number of participants with data at each time point are indicated. (B) IFNγ-ELISpot responses to a peptide library of JEV SA14-14-2 after vaccination. Data are the geometric mean of the sum of spot forming cells (SFC)/10^6^ PBMC for all responding pools (per participant) after subtraction of background wells at each time point ± 95% CI. Non-responding pools were not included. The number of participants is indicated as in (A). Responses were significantly increased over baseline at weeks 1, 2 and 4 (Wilcoxon signed rank test). (C) Proliferation responses in 13 JE vaccinated individuals. Data represent the average % responding cells (CFSE^lo^/CD38^hi^) measured by flow cytometry across all peptide pools tested in the CD4^+^ (filled circles) or CD8^+^ (open circles) gate. Data points are the median and error bars depict the interquartile range (IQR). The number of participants is indicated as in (A).

Two participants vaccinated for occupational reasons were JEV seropositive (PRNT_50_ >1:10) before vaccination. In addition, despite negative screening neutralizing antibody assays using PS cells, two participants on the interventional protocol were found to be seropositive when neutralising antibody measurements were repeated using PRNT_50_ after vaccination. Because NAb at baseline was allowed in the occupational vaccinees, these participants remained in the analysis. Ten out of 16 participants (62.5%) seroconverted to PRNT_50_ >1:10 or >4-fold increase over baseline (Fig 1A and S1 Fig). Of the four seropositive participants at baseline, two seroconverted with 7.2 and 9.7 fold increases in PRNT_50_. Therefore, 12 volunteers were sero-protected after vaccination (75%). Reciprocal geometric mean titre (GMT) at week 4 was 18.5 overall but 51 among those who seroconverted. The reciprocal GMT of maximal responses among seroconverters was 61.5. PRNT_50_ waned after vaccination and, of the nine participants with data 16–26 weeks after vaccination, only four (44%) had PRNT_50_ >1:10.

Participant VA001/1 had a positive IFNγ-ELISpot assay (NS3) at baseline (but was vaccinated because of PRNT_50_ 1:6 and laboratory work with JEV), but made additional responses after vaccination. In total, 13 out of 15 participants tested (87%) developed new IFNγ-ELISpot responses, peaking two weeks after vaccination with significant increases over baseline at weeks 1, 2 and 4 (Fig 1B). However, there was variation amongst the participants with some mounting the peak response later (S1 Fig).

Proliferation responses were available for 13 participants for at least two time points. Interestingly, T cell proliferation responses were detected in most participants before vaccination, despite negative ELISpot assays and/or PRNT_50_. Although five volunteers appeared to make new T cell proliferation responses over the course of the study (S2 Fig), proliferation responses were variable and overall there was no significant difference from baseline values at any time point (Fig 1C). Example flow cytometry data of CFSE assays over the course of the study are shown in supplementary figure S3 Fig.

**Fig 2:**
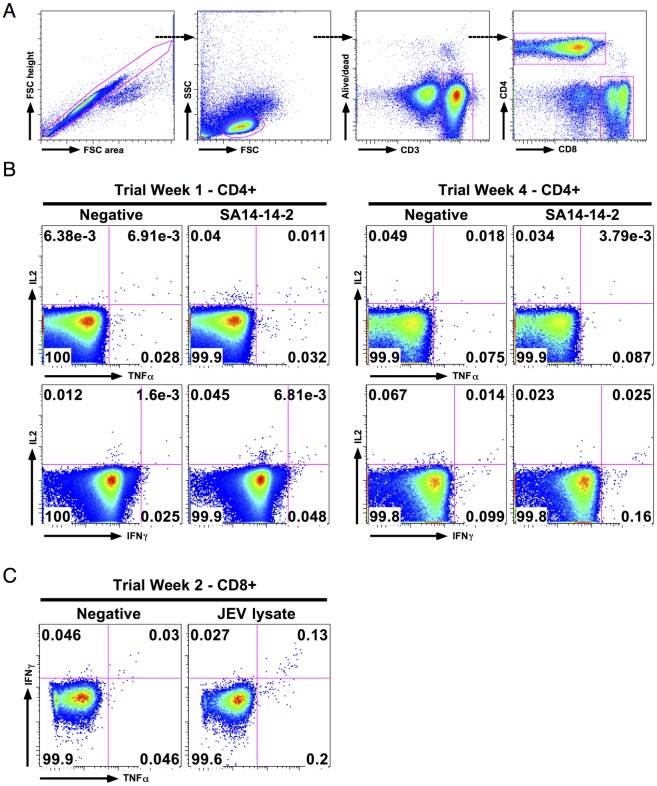
Example data of JE vaccine specific T cell responses by intracellular cytokine staining (ICS). Example flow cytometry data from subject VA019/3, who was JEV NAb positive at baseline, throughout the first four weeks of the study are shown. Data are log_10_ fluorescence units. (A) Example gating strategy; the same strategy was used for all experiments. (B) CD4^+^ T cell responses at trial week 1 and week 4, measured by overnight stimulation of PBMC with approximately 10^4.4^ PFU JEV SA14-14-2. A transient IL2 single positive response was present at week 1 that was no longer seen over the background at week 4. (C) CD8^+^ T cell response to wild type JEV infected cell lysate, used at 80 ng/ml JEV E protein concentration at trial week 2, measured by ICS after stimulation of whole blood for six hours. The CD8^+^ T cell IFNγ response was only detected at week 2, consistent with the peak in ELISpot response (S1 Fig).

**Fig 3:**
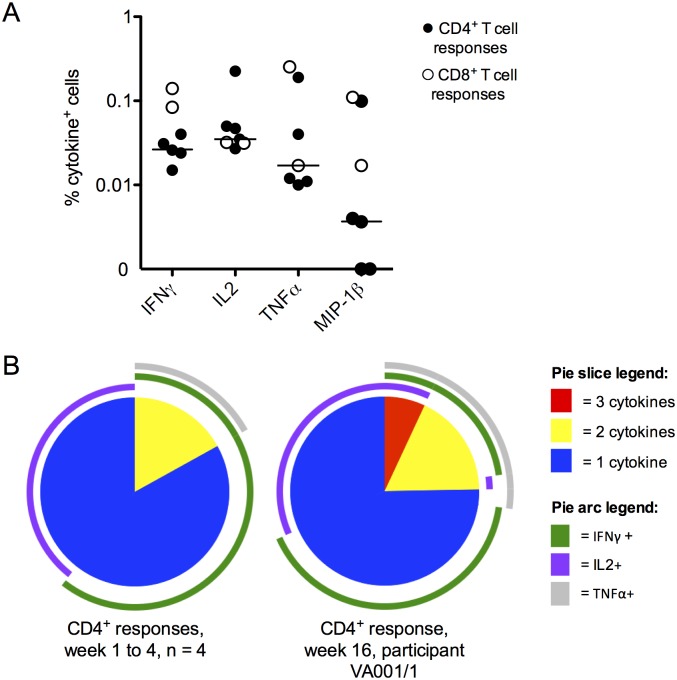
Cytokine production by JE vaccine specific T cells by intracellular cytokine staining (ICS). (A) Magnitude of T cell cytokine responses to JEV SA14-14-2 detected by flow cytometry/ICS in five participants showing positive responses. Data are the percentage of cells in the parent (CD4^+^/CD8^+^) gate staining for the indicated cytokine, above unstimulated values. Filled circles = CD4^+^ responses, open circles = CD8^+^ responses. Bars = median value of CD4^+^ responses only. (B) SPICE analysis of CD4^+^ T cell responses detected by flow cytometry. Data are the proportion of the response comprised of IFNγ^+^/IL2^+^/TNFα^+^ triple positive cells (red pie slices), double positive cells (yellow slices) and single positive cells (blue slices). Arcs indicate the proportion of the response producing individual cytokines: IFNγ (purple arcs), IL2 (green arcs) and TNFα (light grey arcs). Left pie chart: CD4^+^ responses at weeks 1–4 (n = 4), right pie chart: participant VA001/1 at week 16. Participant VA001/1 made a triple positive response at week 16 after vaccination, hence this participant is presented separately.

The function and characteristics of *ex-vivo* T cell responses were investigated further by intracellular cytokine staining (ICS) and flow cytometry in 13 participants with positive ELISpot assays; responses were detected in five (ELISpot is a more sensitive technique than ICS). CD4^+^ T cell responses were detected in three participants; CD4^+^ and CD8^+^ T cell responses in two. Flow cytometry data showing CD4^+^ and CD8^+^ T cell responses throughout the study from participant VA019/3 are shown in Fig 2, though sample limitation meant that we could not perform all these assays on every participant. Cytokine responses were small (Fig 3A) and polyfunctional T cell responses to JE vaccine were rare, in contrast to our recent findings in natural exposure and recovered JE [17]. In CD4^+^ T cell responses, IFNγ^+^ or IL2^+^ only populations dominated (Fig 3B), indicating that IFNγ-ELISpot alone may not detect all such responses. Participant VA001/1 (who showed a CD8^+^ T cell response at baseline) was an exception; we identified a polyfunctional (IFNγ/TNFα/IL2 triple positive) CD4^+^ T cell response at week 16 after vaccination.

### Targeting of IFNγ responses

**Fig 4:**
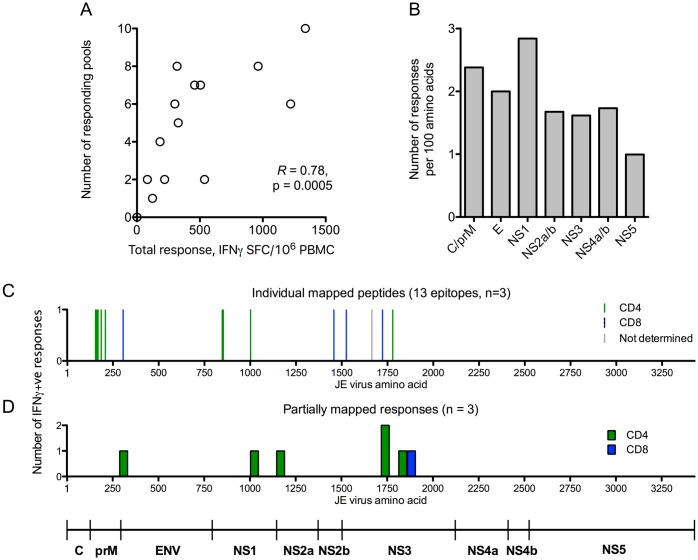
Targeting of IFNγ responses to JEV SA14-14-2. (A) Sum of all responding pools (IFNγ SFC/10^6^ PBMC by ELISpot) against the number of responding pools at the time of maximum ELISpot response per participant in the study. (B) Number of participants responding to each protein of JEV in ELISpot assays, normalised to the protein size as responses per 100 amino acids. (C & D) Peptide pools were deconvoluted by *ex-vivo* ELISpot, or in ICS assays using T cell lines expanded to peptide pools. Location of epitopes mapped to individual peptides (C) and to “mini-pools” spanning regions 46–90 amino acids in size (D). The Y axes represent the number of responses identified (one of the six “mini-pool” regions identified was recognised by two participants). Subsets were determined by flow cytometry. CD4^+^ responses: green bars; CD8^+^ responses: blue bars. The single grey bar in (C) depicts a response identified by ELISpot where the subset was not determined.

Most participants made IFNγ responses to >1 peptide pool (median five) and all viral proteins were targeted. The total magnitude of the ELISpot response correlated with the number of responding pools (Spearman’s *R* = 0.78, p = 0.0005, Fig 4A) indicating that there were no strongly immunodominant pools. Normalised to protein size, NS1 elicited responses most frequently (Fig 4B).

In six participants, further experiments were conducted to identify some of the epitopes recognised after vaccination with JEV SA14-14-2. Responding peptide pools were either mapped *ex-vivo* by ELISpot, or short-term T cell lines (TCL) were expanded to peptide pools showing responses in *ex-vivo* assays. The peptide pools were then deconvoluted first into “mini-pools” of 6–10 peptides which were used to stimulate short term TCL in ICS assays (also allowing determination of the responding subset), followed by mapping down to individual peptides. Fifteen peptides were mapped (Fig 4C and Table 2) and a further six 46 to 90 amino acid regions (Fig 4D and Table 2) eliciting IFNγ responses were identified (participant VA023/1 had a response mapped to mini-pools only). In participant VA019/3 an antigenic region corresponding to amino acids 214–303, in prM, overlapped with peptide TRTRHSKRSRRSVSV, amino acids 209–223. The response to amino acids 214–303 was larger than the response to 209–223, making it unlikely the response to 214–303 was accounted for by only the 10 amino acid overlap. The peptides identified were mostly in the prM, NS1 and NS3 proteins (Fig 4C) with one identified in E protein. Table 2 shows all the peptides and antigenic regions identified.

**Table 2:** Protein sequence of all T cell antigenic regions identified during the study.

	Subject	Peptide sequence	JEV protein	Polyprotein amino acid location	Subset	Changes from genotype III consensus sequence
Mapped peptide responses	VA019/3	SKGENRCWVRAIDVGYM	prM	155–171	CD4	
	VA019/3	WVRAIDVGYMCEDTITY	prM	162–178	CD4	
	VA019/3	GYMCEDTITYECPKL	prM	169–183	CD4	
	VA019/3	GNDPEDVDCWCDNQEVYV	prM	186–203	CD4	
	VA019/3	TRTRHSKRSRRSVSV	prM	209–223	CD4	
	VA020/1	GATWVDLVL	E	306–328	CD8	
	VA019/3	GVCGVRSVTRLEHQMW	NS1	847–862	CD4	
	VA019/3	SVTRLEHQMWEAVRDEL	NS1	853–869	CD4	
	VA019/3	DTWKLERAVFGEVKSCTW	NS1	1002–1019	CD4	
	VA001/1	DFH**F**IDDPGVPWKVWVLR	NS2b	1457–1474	CD8	L1460F
	VA018/1	GILGTYQAGVGVMYENVF	NS3	1525–1542	CD8	
	VA012/3	YVSAIVQGDRQEEPVPEAYTPNM	NS3	1665–1687	ND	
	VA012/3	TAVLAPTRVVAAEMAE**V**L	NS3	1723–1740	CD8	A1739V
	VA001/1	**V**LRGLPVRY (mapped to JEV wt ALRGLPVRY before vaccination)	NS3	1739–1747	CD8	A1739V
	VA001/1	RVPNYNLFVMDEAHF	NS3	1779–1793	CD4	
Mini pools	VA019/3	SKRSRRSVSVQTHGESSLVNKKEAWLDSTKATRYLMKTENWIIRNPGYAFLAAVLGWMLGSNNGQRVVFTILLLLVAPAYSFNCLGMGNR	prM	214–303	CD4	
	VA019/3	EKFEMGWKAWGKSILFAPELANSTFVVDGPETKECPDEHRAWNSMQIEDFGFGITSTRVWLK	NS1	903–964	CD4	
	VA019/3	DYCPGTKVTITEDC**S**KRGPSVRTTTDSGKLITDWCCRSCSLPPLRFRTENGCWYGMEIRPV**M**HDETTLVRSQV**H**AF**K**GEMVDPF	NS1	1072–1155	CD4	G1086S, R1133M, D1145H, N1148K
	VA023/1, VA018/1	PRGTSGSPILDSNGDIIGLYGNGVELGDGSYVSAIVQGDRQEEPVPEAYTPNMLRKRQMTV	NS3	1635–1695	CD4	
	VA023/1	LMSPNRVPNYNLFVMDEAHFTDPASIAARGYIATKVELGEAAAIFM	NS3	1774–1819	CD4	
	VA020/1	ELGEAAAIFMTATPPGTTDPFPDSNAPIHDLQDEIPD**W**AWSSGYEWITEYAGKTV	NS3	1810–1864	CD8	R1847W

Peptide sequences of JEV SA14-14-2 to which T cell responses were identified are shown. The final column and bold/underlined text indicates amino acid differences between SA14-14-2 and a JEV wild type genotype III consensus sequence. ND = not determined.

### Cross-reactivity of IFNγ responses

**Fig 5:**
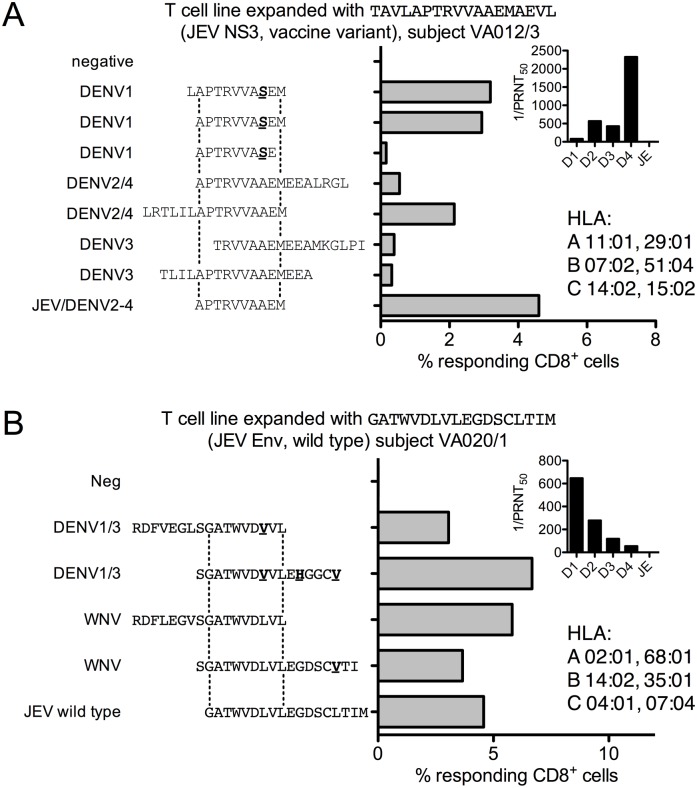
Cross-reactivity of anti-JEV IFNγ responses. (A) cross-reactive T cell responses to variant peptides from dengue virus (DENV) in participant VA012/3. Data are percent IFNγ^+^ CD8^+^ T cells from a short-term T cell line expanded with JE vaccine NS3 peptide TAVLAPTRVVAAEMAEVL in an ICS assay to the peptides indicated. Two earlier experiments expanding TCL to wild type and vaccine library peptides, which differ only in a Val/Ala substitution at position 17, outside the region of epitope conservation (dotted lines), gave the same result. Inset: Reciprocal PRNT_50_ for all four DENV serotypes and JEV; HLA type. (B) cross-reactive T cell responses to variant peptides from West Nile virus and DENV in participant VA020/1. Data are percent IFNγ^+^ CD8^+^ T cells from a short-term T cell line expanded with JE vaccine E peptide GATWVDLVLEGDSCLTIM in an ICS assay to the peptides indicated. The dotted lines indicate the region of epitope conservation subsequently shown in another experiment (S4 Fig panel A). Inset: Reciprocal PRNT_50_ for all four DENV serotypes and JEV; HLA type.

All four DENV serotypes were detected in Karnataka State in the two years prior to this study (S1 Data). Therefore, in some participants, we investigated whether the IFNγ responses identified cross-reacted with DENV. Two participants, VA012/3 and VA020/1, had responses that were not present before vaccination mapped to individual peptides by *ex-vivo* ELISpot assays. Partial peptide libraries from DENV serotypes 1, 3 and 4 and complete libraries from DENV2 and WNV were available (see methods). To test for cross-reactive responses, short term T cell lines (TCL) were expanded by culturing PBMC from the volunteers in the presence of “T-Stim” (IL2), IL7 and the relevant JEV peptide at 10 μg/ml for 7–10 days. The same TCL were then stimulated with JEV peptide alongside variant peptides from DENV or WNV selected on the basis of a ClustalW protein alignment. Because the cells specific for the JEV peptide had expanded in culture (typically from around 0.1% (Fig 3A) to 5% (Fig 5)), a response to the variant peptide of equivalent magnitude to the JEV peptide indicates that it is likely to be the same cells being triggered by the variant peptide and the response is therefore cross-reactive.

Participant VA012/3 had high levels of NAb to DENV4 and intermediate levels of NAb to DENV2 and DENV3 at baseline, and no detectable JEV NAb. This participant developed a CD8^+^ T cell response to peptide TAVLAPTRVVAAEMAEVL (NS3), subsequently mapped to APTRVVAAEM (S4 Fig panel A), very similar to a previously described partially mapped HLA B*07 restricted DENV4 epitope, LAPTRVVAAEME [28]. A short-term T cell line recognised the very close DENV1 sequence APTRVVASEM equally well (Fig 5A); the sequence in DENV2-4 is identical to JEV. Interestingly, this subject did not seroconvert to JE vaccine.

Participant VA020/1, who had the highest titre of NAb to DENV1 with lower levels to DENV2 and DENV3 and no JEV NAb at baseline, developed a CD8^+^ T cell response to GATWVDLVL (E), based on overlapping peptide ELISpot assays and confirmed by flow cytometry with truncated peptides (S4 Fig panel B). This was confirmed by expanding a short-term TCL (Fig 5B); the epitope cross-reacted with a variant peptide conserved in DENV1 and DENV3. In a subsequent experiment variant peptides from DENV2 and DENV4 were not recognised, consistent with the serology assays suggesting DENV1 exposure (S4 Fig panel C). These responses represent either priming by JE vaccine that cross-reacts with DENV, or priming by DENV, boosted by JE vaccine, that cross-reacts with additional DENV serotypes because of close sequence similarity.

Participant VA019/3 was DENV exposed (DENV2 PRNT_50_ 1:538), but also had JEV NAb (titre 1:123) before vaccination. This participant had several CD4^+^ responses mapped by expanding short term TCL to responding pools in the ELISpot assays using 5 μg/ml equivalent concentration of each peptide. The short term TCL were then stimulated with smaller pools (“mini-pools”) followed by deconvolution down to individual peptides (Table 2), Finally, the same TCL were then tested with variant peptides from DENV and WNV. As before, because TCLs were expanded with JEV peptides, responses to variant peptides indicate cross-reactivity, though in this case all but one of the responses were JEV specific (Table 3).

**Table 3:** Summary of cross reactive T cell responses identified after vaccination with JEV SA14-14-2.

Subject	Peptide sequence	JEV protein	Polyprotein amino acid location	Subset	Cross-reactive with DENV or WNV
VA019/3	SKGENRCWVRAIDVGYM	prM	155–171	CD4	No
VA019/3	WVRAIDVGYMCEDTITY	prM	162–178	CD4	No
VA019/3	GYMCEDTITYECPKL	prM	169–183	CD4	No
VA019/3	GNDPEDVDCWCDNQEVYV	prM	186–203	CD4	No
VA019/3	TRTRHSKRSRRSVSV	prM	209–223	CD4	No
VA020/1	GATWVDLVL	E	306–328	CD8	Yes (DENV1/3, WNV)
VA019/3	GVCGVRSVTRLEHQMW	NS1	847–862	CD4	Yes (DENV4)
VA019/3	SVTRLEHQMWEAVRDEL	NS1	853–869	CD4	Yes (WNV)
VA019/3	DTWKLERAVFGEVKSCTW	NS1	1002–1019	CD4	No
VA001/1	DFH**F**IDDPGVPWKVWVLR	NS2b	1457–1474	CD8	ND
VA012/3	YVSAIVQGDRQEEPVPEAYTPNM	NS3	1665–1687	ND	ND
VA012/3	TAVLAPTRVVAAEMAE**V**L	NS3	1723–1740	CD8	Yes (DENV1-4, WNV)
VA001/1	**V**LRGLPVRY	NS3	1739–1747	CD8	Yes (DENV2/4, WNV)
VA001/1	RVPNYNLFVMDEAHF	NS3	1779–1793	CD4	ND

Cross-reactive responses to peptide sequences identified in JEV SA14-14-2 are shown. Amino acid differences between SA14-14-2 and JEV genotype III consensus are bold/underlined. ND = not determined, WNV = West Nile virus.

**Fig 6:**
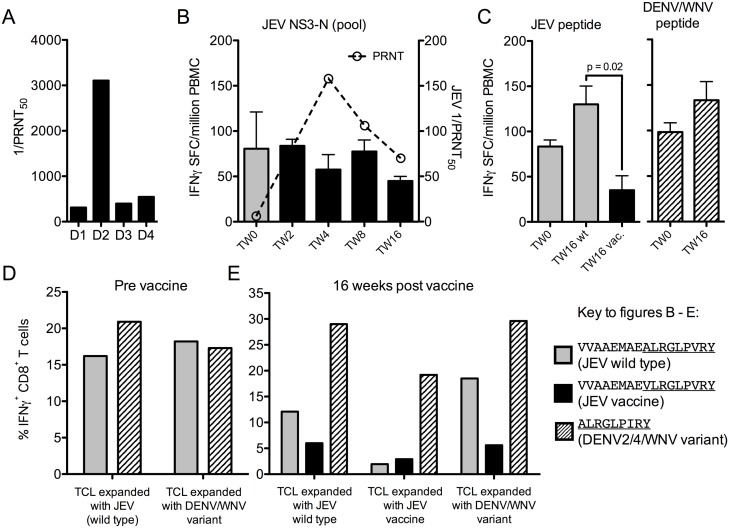
Sub-optimal cross-reactivity of anti-JEV vaccine IFNγ responses in a DENV exposed participant. (A) Reciprocal DENV PRNT_50_ were measured for all four DENV serotypes by plaque assay on MK_2_ cells. (B) NS3 peptide pool IFNγ-ELISpot and NAb responses during the course of the study. (C) IFNγ-ELISpot responses to individual peptides at baseline and 16 weeks. Grey bars = wild type pools or peptide (VVAAEMAEALRGLPVRY), black bars = vaccine pools or vaccine peptide (VVAAEMAEVLRGLPVRY), hatched bars = DENV/2/4WNV peptide (VVAAEMAEALRGLPIRY before vaccination, ALRGLPIRY after vaccination). ELISpot data are presented after subtraction of background values; all responses shown met the criteria for a positive assay (>50 IFNγ-SFC/10^6^ PBMC and double the negative control). The IFNγ-ELISpot response to the JE vaccine peptide was significantly smaller than the response to the wild type peptide at the end of the study (paired t-test). (D) IFNγ responses (percent IFNγ^+^ CD8^+^ T cells) of short-term T cell lines (TCL) expanded with JEV and variant peptides before vaccination, and (E) IFNγ responses of TCL 16 weeks after vaccination, tested against both JEV and variant peptides. The peptides used before vaccination were VVAAEMAEALRGLPVRY (JEV) and VVAAEMAEALRGLPIRY (WNV, epitope in common with DENV2/4). After vaccination, TCL were expanded with minimal peptides ALRGLPVRY (JEV) and ALRGLPIRY (DENV2/4, WNV) but tested against library peptides VVAAEMAEALRGLPVRY (wild type JEV) and VVAAEMAEVLRGLPVRY (JE vaccine) in addition to ALRGLPIRY (DENV2/4, WNV).

**Fig 7:**
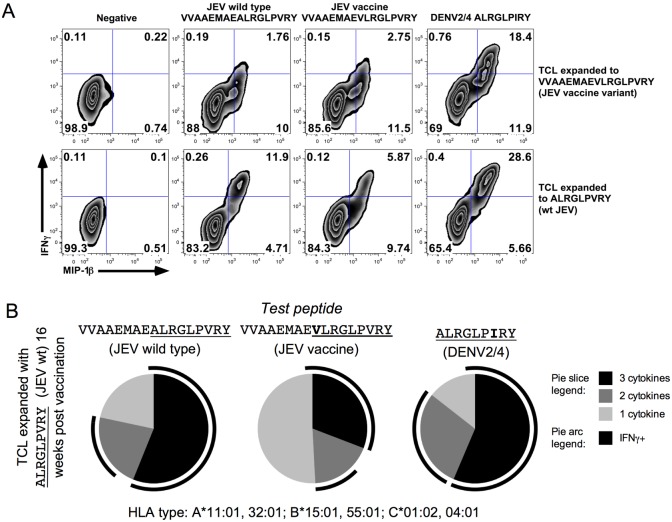
A sub-optimal cross-reactive CD8^+^ T cell response dominated by MIP-1β single positive cells. (A) Flow cytometry data from the same experiment as Fig 6E. Data are log_10_ fluorescence units, gated on live, CD3^+^, CD8^+^ cells. (B) SPICE analysis of 16-week post vaccine TCL responses from the same experiment as in Fig 6E, stimulated with JEV wild type (left), JEV vaccine (centre) and DENV2/4 (right) peptides. Cells were stained for IFNγ, TNFα and MIP-1β; pie slices correspond to the relative proportion of the response made up of triple cytokine^+^ cells (black), double cytokine^+^ cells and single cytokine^+^ cells, which were exclusively MIP-1β^+^ in this experiment.

Participant VA001/1 carries the HLA B*15:01 allele and had high levels of DENV2 NAb and intermediate levels to other serotypes at baseline (Fig 6A). Peptide ALRGLPVRY was mapped before vaccination, and a cross-reactive response was identified to the previously described HLA-B*15 restricted peptide ALRGLP**I**RY from DENV2/4 and WNV [29]. The corresponding vaccine library peptide VVAAEMAE**V**LRGLPVRY had a modest Val for Ala substitution corresponding to position 1 of the 9-mer (the same position as the NS3 peptide TAVLAPTRVVAAEMAE**V**L recognised by participant 012/3), and is predicted to bind the same HLA allele as the wild type peptide with slightly lower affinity (IEBD.com [30, 31]). The *ex-vivo* IFNγ response to the pool containing this peptide did not change after vaccination or with seroconversion (Fig 6B), nor did the responses to the wild type and DENV peptides at week 16 (Fig 6C), though other responses developed. The SA14-14-2 peptide produced a smaller IFNγ response, though the response was still detectable (Fig 6C left panel). Short-term T cell lines expanded with JEV wild type peptide before and after vaccination (Fig 6D & 6E) confirmed that IFNγ responses were smaller to the vaccine peptide (Fig 6E). However, when analysed for additional cytokines, the total number of responding cells was similar, with the difference mostly accounted for by MIP-1β single positive cells (Fig 7A & 7B). MIP-1β may have a lower triggering threshold than other cytokines [32], suggesting that the vaccine variant epitope is less efficient than the wild type in this case. This represents an example where responses to DENV2/4 and JEV are highly cross-reactive with each other, but were less efficiently cross-reactive with the SA14-14-2 variant.

Together, these data show that T cell responses induced to JEV vaccine SA14-14-2 can recognise wild type JEV, and that responses primed to natural flavivirus infection can cross-react with JEV vaccine SA14-14-2 in some instances. These data are consistent with our recent finding that CD8^+^ T cell responses to JEV are highly cross-reactive, whereas CD4^+^ responses are much less so [17], although the small number of participants in the present study prevents generalisation of these findings.

## Discussion

We have shown that, in participants resident in a JE endemic area, T cell IFNγ responses are detectable after vaccination with JEV SA14-14-2, are modest in magnitude, peak within eight weeks of vaccination, and return to baseline levels by 4–6 months. Determination of epitope specificity and cross-reactivity in four participants showed that some responses to JE vaccine can cross-react with DENV, and in one case a variant epitope of SA14-14-2 was recognised less efficiently than the JEV wild type peptide by memory CD8^+^ T cells from a pre-existing response.

Our study found a seroconversion rate of 62.5%, sero-protection rate of 75% and GMT of 18.5 four weeks after vaccination with a single dose of JEV SA14-14-2. This is lower than studies of single dose JEV SA14-14-2 in children, where the seroconversion rate is 80–99% and GMTs 4 weeks after vaccination range from 56–370 [7, 8, 33]; or even higher after previous JEV exposure or vaccination in a DENV non-endemic area (Korea) [34]. However, our findings are consistent with a recently published clinical trial using SA14-14-2 in India in which 57.7% of participants seroconverted at 4 weeks, including 54.9% of adults aged 18–50, a similar age group to this study [35]. In the study of Singh *et al*., seroconversion in children aged 1–6 years was 58.8%, suggesting it is the environment and not age driving this effect. The greater number of participants showing T cell responses than NAb, and the presence of proliferation responses at baseline, indicate that measuring NAb alone may not be the only potential test of JE-immunity. NAb titres to JE vaccines can fall below “protective” levels after vaccination in the presence of protection and rapid recall responses [36, 37], a feature also observed following HBV vaccination, where B cell memory pools may be detected [38].

DENV circulates in South India where this study was conducted. Four participants in whom DENV PRNT_50_ were measured all showed neutralising antibody to DENV, indicative of past infection, as did a larger group recently recruited in Karnataka State, South India [17]. One possibility is that immune interference by DENV may account for the lower seroconversion rate after JEV SA14-14-2 in Indian adults. Experiments are underway to investigate the possibility of interference by DENV exposure. So far, five more participants have been tested for DENV3 NAb post JE vaccine, including two participants who did not seroconvert. Preliminary data suggest our study population is highly DENV exposed, and we have so far not identified any evidence of an increase in DENV NAb titres after JE vaccine, or an original antigenic sin type response.

Cross-reactive T cells against DENV are readily detectable after natural infection or attenuated tetravalent dengue vaccine (TV003) [32, 39] and have been associated with pathology [40] but also protection [41, 42]. One hypothesis for the disappointing results with the new dengue vaccine, Dengvaxia, in some studies [43, 44] is that, because the vaccine is a chimera based on yellow fever 17D, the appropriate T cell responses are not elicited [14]. In this study, as well as in our earlier work [17], we have observed a high degree of T cell cross-reactivity between JEV and dengue viruses in adults in this JE/dengue co-endemic area. This may reflect priming by multiple flavivirus exposures, whereby the most conserved epitopes, which receive the largest number of re-stimulations, are the most readily detectable. This is consistent with observations in a humanised animal model and following tetravalent vaccination [39, 45]. However, this is not always seen in dengue endemic areas, where a sizeable fraction of the T cell response is directed against serotype specific epitopes even in populations with multiple DENV exposures [46]. Conservation between JEV and DENV epitopes and the cross-reactivity observed in most individuals who were tested here suggests that a better understanding of how such responses develop and whether they are protective would be of benefit. There may be strategies that could include such epitopes in next generation vaccines, such as a chimeric JEV/DENV vaccines [47, 48], or heterologous prime/boost immunisation schedules with dengue and JE vaccines.

The *ex-vivo* T cell cytokine profiles after vaccination in this study were different from those seen in our recent study of circulating memory T cell responses to JEV [17]. In our previous study, 75% of responding CD8^+^ T cell responses in healthy, JEV exposed people made two or more cytokines; CD4^+^ T cell responses were very infrequent. Compared with recovered JE patients, where CD4^+^ T cell responses were much more frequent, CD4^+^ T cell responses in this study showed many fewer TNFα secreting cells and cells making two or more cytokines; instead IFNγ and IL2 single positive cells dominated the response. High TNFα levels have been linked to mortality in JE [49], and this finding provides further support for our earlier observations linking CD4^+^ T cell derived TNFα with pathogenesis in JE.

JE vaccination is effective even if the vaccine virus is relatively genetically distant from circulating virus [5] and protection may be long lasting even in the absence of NAb [50]. In our study, 10 individuals were NAb negative at baseline and had proliferation data available. Nine had T cell proliferative responses at baseline, reflective of ‘central’ memory (which presumably also make cytokines, though we did not measure this) rather than IFNγ-ELISpot assays, which, by virtue of their much shorter incubation period, are biased towards ‘effector’ memory [51]. The combination of proliferation in the absence of NAb and ELISpot responses may reflect a common, but hitherto unrecognised, state of long term immunity, mediated by long lived memory T cells [52].

In summary, here we have given the first description of T cell responses to JE vaccine SA14-14-2 in adults in a flavivirus endemic area, most of whom have evidence of prior adaptive immunity against JEV. We have shown that (i) T cell responses were detected in most volunteers after vaccination and cross-react with other flaviviruses; (ii) seroconversion after vaccination of adults with single dose JEV SA14-14-2 in an endemic area is relatively poor and (iii) T cell proliferative responses were detectable before vaccination in most volunteers, even if ELISpot and NAb assays were negative. T cell proliferation is worthy of investigation, in JEV naïve subjects, as an additional immunologic measure of response to vaccine [53]. To what extent prior exposure to related flaviviruses and associated T cell cross reactivity could influence vaccine responsiveness, especially in the B cell compartment, requires further study. Some of the questions surrounding the nature of the response to JEV SA14-14-2 can best be addressed in populations without extensive prior flavivirus exposure.

## Supporting Information

S1 Fig Individual data from IFNγ-ELISpot and neutralising antibody assays. Data for each individual in the study who had ELISpot assays performed are shown (solid lines/black circles), along with PRNT_50_ values (dotted lines/open circles). One participant, VA010/3, did not have ELISpot assays performed, this subject also did not have any neutralising antibody detected when sampled at week 8 post vaccination, hence this participant is not shown. Data are IFNγ spot forming cells (SFC)/10^6^ PBMC on the left hand Y axes, and reciprocal PRNT_50_ measurements on the right hand axes.
(TIF)

S2 Fig Individual data from proliferation assays. Data for each individual in the study who had proliferation assays performed are shown for CD4^+^ T cells (solid lines/black circles) and CD8^+^ T cells (dotted lines/open circles). Data are average percent responding cells (CFSE^lo^/CD38^hi^) measured by flow cytometry across all peptide pools tested in the CD4^+^ or CD8^+^ gate.
(TIF)

S3 Fig Example flow cytometry data from CFSE proliferation assays. PBMC were labelled with CFSE and cultured for eight days in the presence of 3 μg/ml JEV peptide pools. Data for CD4^+^ T cells from participant VA020/1 pre-vaccination and at weeks 2 and 16 are shown.
(TIF)

S4 Fig Further mapping and cross-reactivity data for participants VA012/3 and VA020/1. (A) A short term T cell line was expanded from participant VA012/3 to JEV vaccine peptide TAVLAPTRVVAAEMAEVL, which differs from the wild type JEV peptide by a Val for Ala substitution at position 17, was tested against the truncated peptides shown. (B) A short term T cell line was expanded from participant VA020/1 to JEV peptide GATWVDLVLEGDSCLTIM and tested against the truncated peptides shown. The response was mapped to GATWVDLVL. Data are the percentage of responding CD8^+^ T cells in an IFNγ/TNFα ICS assay. (C) A short term T cell line was expanded to JEV peptide GATWVDLVL and tested against the DENV variants shown. Although this line did not expand very well, and the cross-reactive response to the DENV1/3 peptide is less than Fig 5B, it meets the criteria for a positive response. No response was seen to peptides of DENV2 or DENV4. Data are the percentage of responding CD8^+^ T cells in an IFNγ/TNFα ICS assay.
(TIF)

S1 JEV Peptide library (XLS)

S1 Data Dengue virus serotype specific RT-PCR data. (DOCX)

S2 Data Study dataset. (XLSX)

S1 Protocol The protocol is for the interventional study, participants being vaccinated for occupational reasons followed an identical protocol, except for pre-vaccination screening. (PDF)

S1 TREND checklist (PDF)
