# Supplementary figures and images for: Cellular Immune Responses to Live Attenuated Japanese Encephalitis (JE) Vaccine SA14-14-2 in Adults in a JE/Dengue Co-Endemic Area
Source: PLoS Negl Trop Dis. 2017 Jan 30;11(1):e0005263. doi: 10.1371/journal.pntd.0005263 (PMC5279729; doi:10.1371/journal.pntd.0005263)

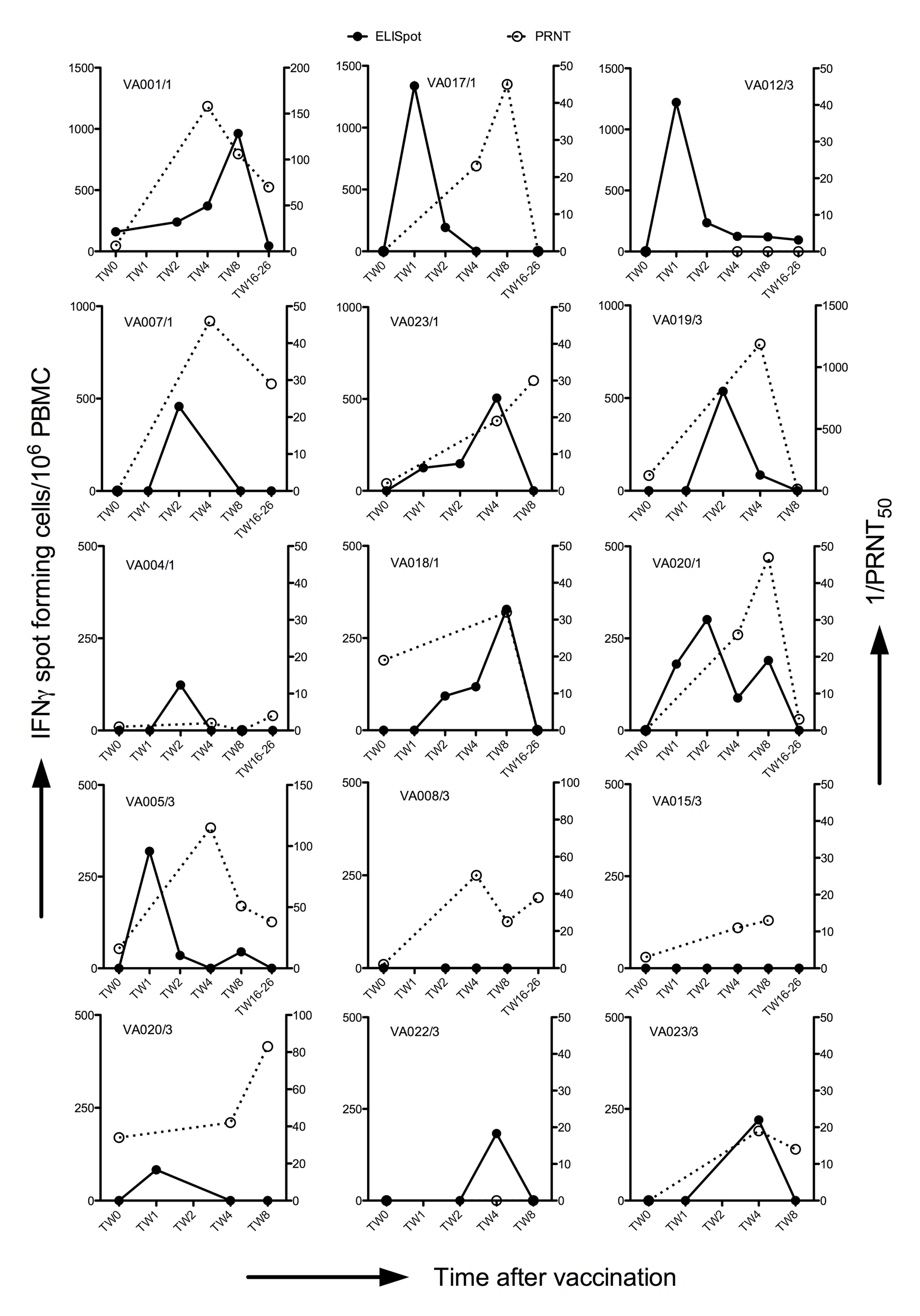

Supplement: S1 Fig — Data for each individual in the study who had ELISpot assays performed are shown (solid lines/black circles), along with PRNT50 values (dotted lines/open circles). One participant, VA010/3, did not have ELISpot assays performed, this subject also did not have any neutralising antibody detected when sampled at week 8 post vaccination, hence this participant is not shown. Data are IFNγ spot forming cells (SFC)/106 PBMC on the left hand Y axes, and reciprocal PRNT50 measurements on the right hand axes. (TIF) [file pntd.0005263.s001.tif]

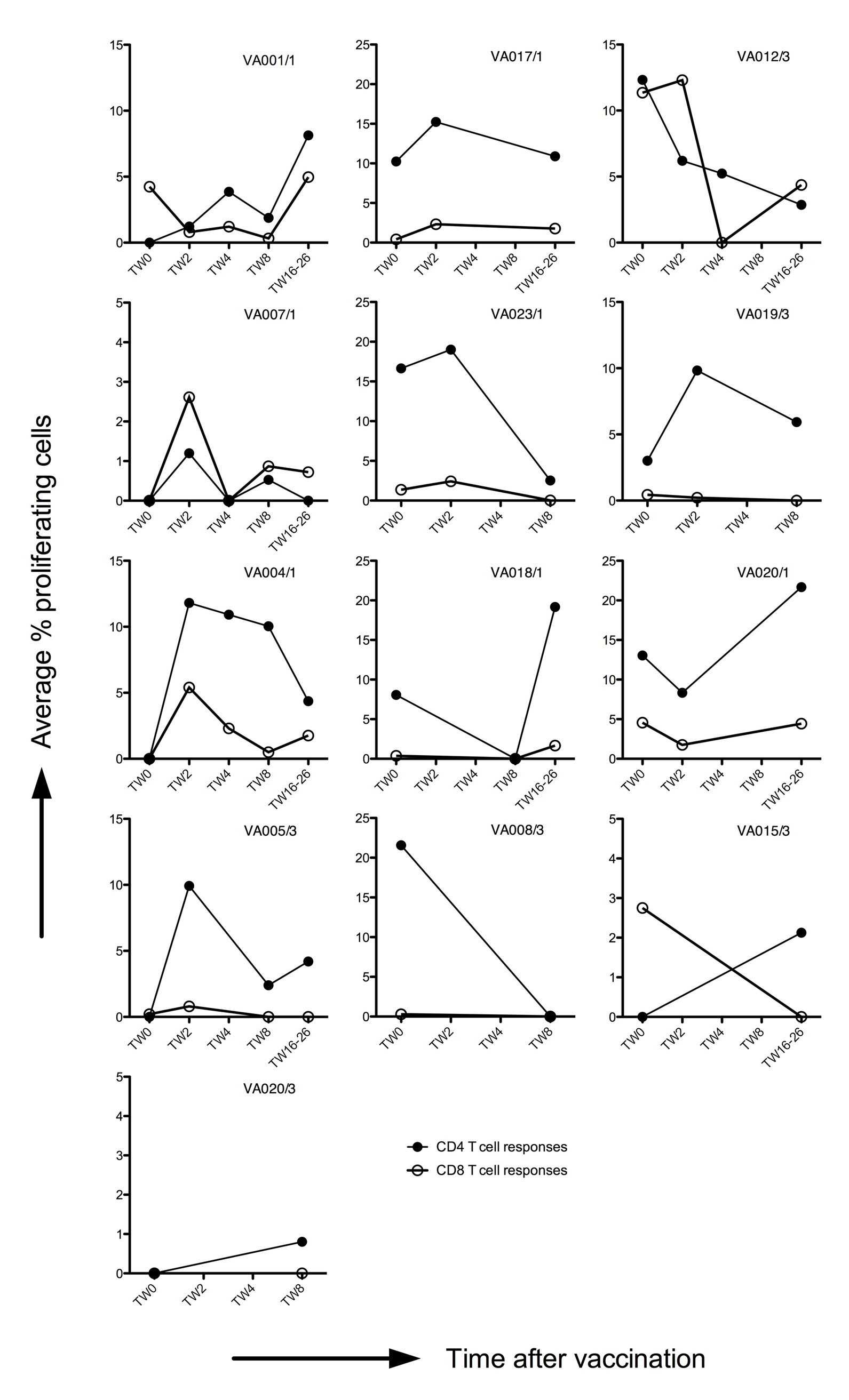

Supplement: S2 Fig — Data for each individual in the study who had proliferation assays performed are shown for CD4+ T cells (solid lines/black circles) and CD8+ T cells (dotted lines/open circles). Data are average percent responding cells (CFSElo/CD38hi) measured by flow cytometry across all peptide pools tested in the CD4+ or CD8+ gate. (TIF) [file pntd.0005263.s002.tif]

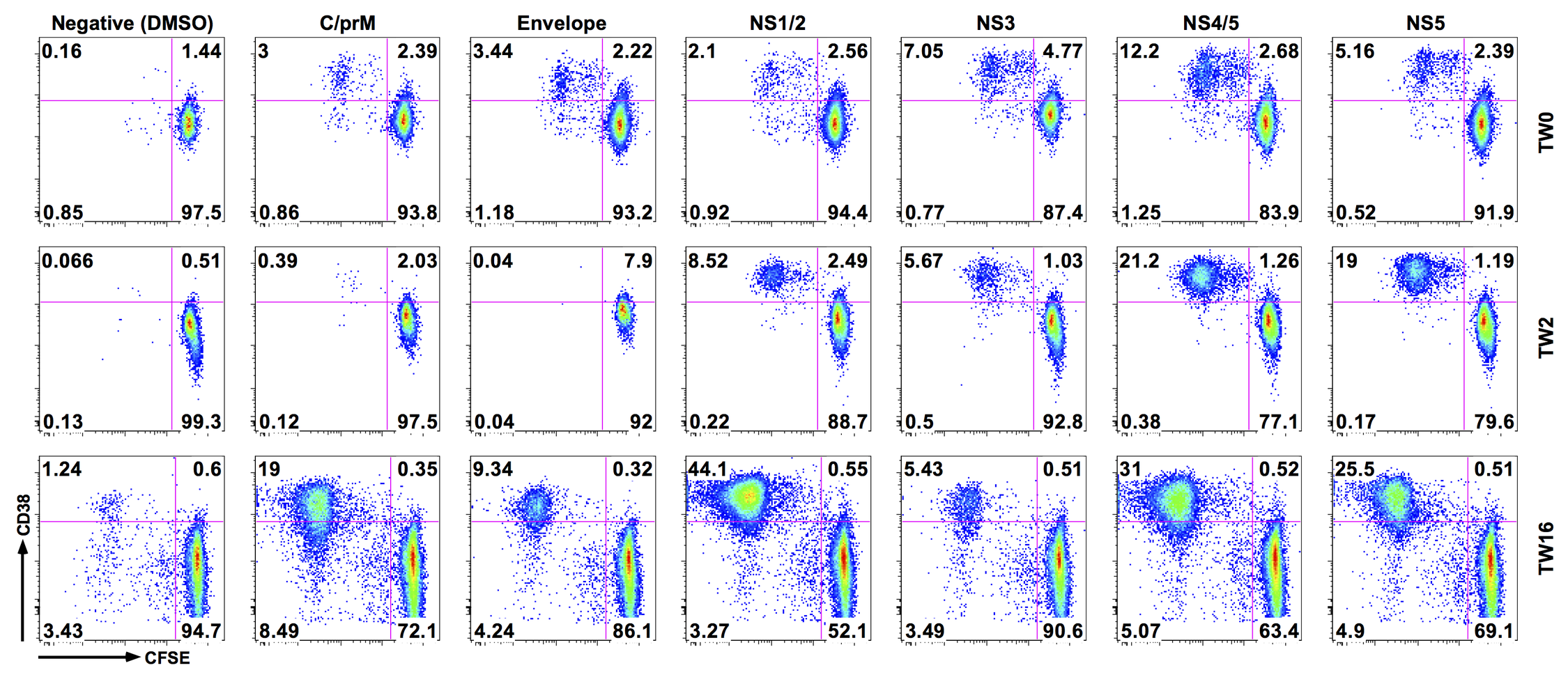

Supplement: S3 Fig — PBMC were labelled with CFSE and cultured for eight days in the presence of 3 μg/ml JEV peptide pools. Data for CD4+ T cells from participant VA020/1 pre-vaccination and at weeks 2 and 16 are shown. (TIF) [file pntd.0005263.s003.tif]

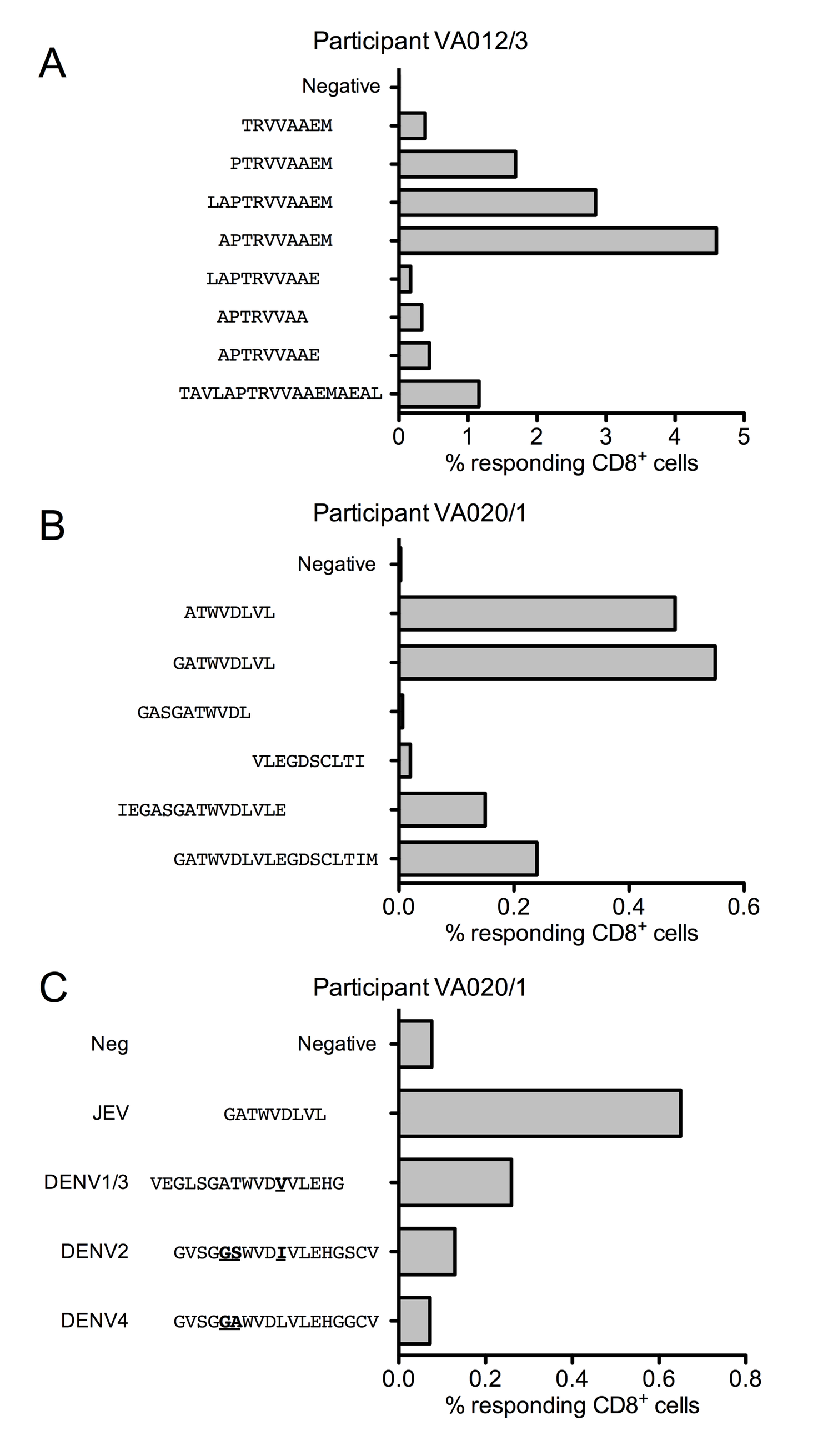

Supplement: S4 Fig — (A) A short term T cell line was expanded from participant VA012/3 to JEV vaccine peptide TAVLAPTRVVAAEMAEVL, which differs from the wild type JEV peptide by a Val for Ala substitution at position 17, was tested against the truncated peptides shown. (B) A short term T cell line was expanded from participant VA020/1 to JEV peptide GATWVDLVLEGDSCLTIM and tested against the truncated peptides shown. The response was mapped to GATWVDLVL. Data are the percentage of responding CD8+ T cells in an IFNγ/TNFα ICS assay. (C) A short term T cell line was expanded to JEV peptide GATWVDLVL and tested against the DENV variants shown. Although this line did not expand very well, and the cross-reactive response to the DENV1/3 peptide is less than Fig 5B, it meets the criteria for a positive response. No response was seen to peptides of DENV2 or DENV4. Data are the percentage of responding CD8+ T cells in an IFNγ/TNFα ICS assay. (TIF) [file pntd.0005263.s004.tif]
