# Supplementary material for: Cellular Immune Responses to Live Attenuated Japanese Encephalitis (JE) Vaccine SA14-14-2 in Adults in a JE/Dengue Co-Endemic Area
Source: PLoS Negl Trop Dis. 2017 Jan 30;11(1):e0005263. doi: 10.1371/journal.pntd.0005263 (PMC5279729; doi:10.1371/journal.pntd.0005263)
Supplement: S1 Data — (DOCX) [file pntd.0005263.s006.docx]

**Supplementary data – dengue virus serotype specific RT-PCR**

In the period 2010 to 2011, shortly before this study commenced, 98 serum samples from suspected dengue cases from sites in Karnataka State were subject to serotype specific PCR. These assays were performed in the NIMHANS diagnostic laboratory, as part of the National vector borne disease control program using the method of Shu *et al* [1]. All four dengue virus serotypes were identified in these samples.

| **Dengue serotype** | **Number RT-PCR positive** |
| --- | --- |
| DENV1 | 10 |
| DENV2 | 13 |
| DENV3 | 2 |
| DENV4 | 7 |
| DENV1 & 2 | 3 |
| DENV2 & 4 | 2 |
| Negative | 61 |

**Supplementary Table. Dengue virus serotype specific RT-PCR.**

1. Shu PY, Chang SF, Kuo YC, Yueh YY, Chien LJ, Sue CL, et al. Development of group- and serotype-specific one-step SYBR green I-based real-time reverse transcription-PCR assay for dengue virus. J Clin Microbiol. 2003;41(6):2408-16.
