# Supplementary material for: Cellular Immune Responses to Live Attenuated Japanese Encephalitis (JE) Vaccine SA14-14-2 in Adults in a JE/Dengue Co-Endemic Area
Source: PLoS Negl Trop Dis. 2017 Jan 30;11(1):e0005263. doi: 10.1371/journal.pntd.0005263 (PMC5279729; doi:10.1371/journal.pntd.0005263)
Supplement: S1 Protocol — (PDF) [file pntd.0005263.s008.pdf]

CLINICAL TRIAL PROTOCOL

Title	A prospective, open label study of human T cell responses to live attenuated Japanese Encephalitis vaccine SA14-14-2.
Protocol no	JEV SA14-14-2/T cell/01
Version no & date	Version 7, 20th September 2011
Phase	IV
Funded by	Wellcome Trust of Great Britain
Sponsor	Indian Institute of Science, Bangalore
Investigational product	Japanese encephalitis live attenuated SA14-14-2 vaccine

INVESTIGATOR AGREEMENT

This trial will be conducted according to the ICH tripartite guidelines on Good Clinical Practice (see http://www.ich.org/pdfICH/e6.pdf), according to this Clinical Trial Protocol and according to Indian regulatory requirements.

I agree:

- To assume responsibility for the proper conduct of the study at this site
- To conduct the study in compliance with this protocol, any mutually agreed future protocol amendments, and with any other study conduct procedures provided by the sponsor(s).
- Not to implement any changes to the protocol without agreement from the sponsors and prior review and written approval from the relevant study ethical review committees, except where necessary to eliminate an immediate hazard to the participants, or for administrative aspects of the study (where permitted by all applicable regulatory requirements).
- That I am thoroughly familiar with the appropriate use of the vaccine, as described in this protocol, and any other information provided by the sponsors, including but not limited to, the following: the current Investigator's Brochure (IB).
- That I am aware of, and will comply with, ICH and current Good Clinical Practices and all applicable regulatory requirements

CONTACT DETAILS

Principal investigator: Prof S Vijaya
Microbiology & Cell Biology
Indian Institute of Science
CV Raman Avenue
Bangalore 560012
+91 80 2293 2685
vijaya@mcbl.iisc.ernet.in

Principal investigator: Prof V Ravi
Head of Neurovirology & Registrar
NIMHANS
Hosur Road
Bangalore 560029
+91 80 2699 5126
virusravi@gmail.com

Investigator: Dr Lance Turtle
Microbiology & Cell Biology
Indian Institute of Science
CV Raman Avenue
Bangalore 560012
+91 99023 19005
lance.turtle@liverpool.ac.uk

Study Coordinator: Dr Sajesh TK
Microbiology & Cell Biology
Indian Institute of Science
CV Raman Avenue
Bangalore 560012
+91 99168 70804
Sajeshtk.tk@gmail.com

Collaborator: Dr Mansour Yaïch
Vaccine Development Director

Partners for Appropriate Technologies in Health (PATH)
Bâtiment Avant Centre
13 chemin du Levant
01210 Ferney Voltaire
France
+33 233 30 276 6154
myaich@path.org

Collaborator: Prof Tom Solomon
Director, Institute of Infection & Global Health;
Head, Brain Infections Group
University of Liverpool
8th Floor, Duncan Building
Daulby Street
Liverpool L69 3GA
+44 151 706 4603
tsolomon@liverpool.ac.uk

Collaborator: Prof Paul Klenerman
Wellcome Trust Senior Clinical Fellow
University of Oxford
Peter Medawar Building for Pathogen Research
South Parks Road
Oxford OX1 3SY
+44 1865 281885
paul.klenerman@medawar.ox.ac.uk

TABLE OF CONTENTS **PAGE NUMBER**

**1. SYNOPSIS**	7
1.1 Study title	7
1.2 Trial site	7
1.3 Trial Period	7
1.4 Study Population	7
1.5 Objectives	7
1.6 Methodology	7
1.7 Endpoints	9
1.8 Statistical Considerations	10
**2. BACKGROUND AND INTRODUCTION**	11
2.1 Japanese encephalitis	11
2.2 Epidemiology of JE in India	11
2.3 Human Immune response to JEV	12
2.4 Vaccines against JE	13
2.5 Brief description of the vaccine SA14-14-2	14
2.6 Characterisation of live JE vaccine SA14-14-2	15
2.7 Specific Pharmacological actions	18
2.8 Summary of clinical safety studies	21
2.9 Rationale for the study	21
**3. METHODS**	23
3.1 Study design	23
3.2 Screening and visit	26
3.3 Concomitant medications	28
3.4 Procedure for monitoring subject compliance	28
**4. SUMMARY OF STUDY REQUIREMENTS**	29
4.1 Investigational material	29
4.2 Reconstitution and Administration of the Vaccine	29
4.3 Vaccine accountability	30
**5. ASSESMENT OF SAFETY**	31
5.1 Safety Monitoring	31
5.2 Discontinuation criteria/Stopping rules	31
5.3. Adverse Events	31
5.4 Assessment of Causality	32
5.5 Treatment of Adverse events	33
5.6 Adverse Event Follow-up & Assessment of Outcome	33
5.7 Pregnancy	34

6. LABORATORY 35

6.1 Haematology/Biochemistry/Urinalysis 35
6.2 Serology 35
6.3 Specimen handling and analysis 35
6.4 Immunological Experiments 35

7. ETHICAL CONSIDERATIONS 37

7.1 Consent 37
7.2 Risks 37
7.3 Benefits 38
7.4 HIV testing 39

8. QUALITY CONTROL AND QUALITY ASSURANCE 40

9. CONFIDENTIALITY 41

9.1 Direct access to source data/documents 41
9.2 Data handling and record keeping 41

10. FINANCING 42

11. PUBLICATION POLICY 42

1. SYNOPSIS

1.1 Study Title

A prospective open label study of human T cell responses to live attenuated Japanese encephalitis vaccine SA14-14-2.

1.2 Trial sites

- Indian Institute of Science, Bangalore 560012
- Dept. of Neurovirology, NIMHANS, Bangalore 560029

1.3 Trial period

May 2012 – May 2013

1.4 Study population

Healthy adults aged 18 – 50

1.5 Objectives

1.5.1 Primary objective

- To describe the nature, quality and duration of the human T cell response to live attenuated Japanese encephalitis (JE) vaccine SA14-14-2.

1.5.2 Secondary objectives

- To determine seroconversion to the vaccine at one month, six months and one year after vaccination.
- To determine the occurrence of adverse events after vaccination.

1.6 Methodology

Open label, non-controlled trial

Eligible participants will be vaccinated with injectable live attenuated JE Virus (JEV SA14-14-2).

Participants will have contact with a study doctor to report adverse events.

1.6.1 Number of participants planned

Total number of participants:	20
Expected drop out rate:	25%
Approximate number to be enrolled:	25
Expected screen failures:	80%

Prospective participants will be screened until a sufficient number of eligible participants are identified. We aim to have approximately equal numbers of dengue virus (DENV) exposed and unexposed individuals.

1.6.2 Inclusion criteria:

- A male or female adult between 18 and 50 years of age.
- Written informed consent.
- Free of obvious health problems as established by medical history and history-directed physical examination before entering the study.
- Expected continuous residence in India during study period, without travel outside India
- An efficacious method of contraception must be used during the study for women of childbearing potential.

1.6.3 Exclusion criteria:

- Use of any investigational or non-registered drug or vaccine other than the study vaccine within 30 days preceding administration of SA14-14-2 vaccine, or planned use during the study period.
- Chronic administration of immunosuppressants or other immune-modifying drugs within a period of six months before vaccination or at any time during the study period.
- Any confirmed or suspected immunosuppressive or immunodeficient condition.
- A family history of congenital or hereditary immunodeficiency.
- Any antiviral drug therapy within a period of six months before vaccination or at any time during the study period.
- History of significant allergic disease or reactions likely to be exacerbated by any component of the study vaccine, especially allergic disease or reactions to any previous dose of any vaccine.
- History of having received JE vaccine, yellow fever vaccine, tick-borne encephalitis vaccine or experimental flavivirus vaccine.
- History of documented JE infection.
- Detectable anti JE or West Nile neutralizing antibodies in screening tests.

- Acute disease at the time of enrollment. Entry into the study may be deferred until the illness is resolved.
- Acute or chronic, clinically significant, pulmonary, cardiovascular, hepatic, neurological or renal functional abnormality, as determined by history and physical or laboratory examination that is not controlled by drugs.
- Administration of immunoglobulins and/or any blood products within the three months preceding administration of vaccine, or planned administration during the study period.
- Seropositive for HIV, HCV or HbsAg.
- Lactation, pregnancy or intention to get pregnant.
- History of excessive alcohol consumption, drug abuse or significant psychiatric illness.
- Any other condition that in the opinion of the investigator would pose a health risk to the participant or interfere with the evaluation of the vaccine.

1.7 Endpoints

1.7.1 Primary endpoint

The primary endpoint of this study is a description of the T cell immune response to the live attenuated JE vaccine SA14-14-2. Peripheral blood mononuclear cells (PBMC), which include peripheral blood T cells, will be stimulated *in vitro* using a synthetic peptide library covering the entire genome of the virus. This will allow the determination of where within the virus the important T cell antigens reside, according to HLA type. Markers of activation, proliferation, apoptosis and immune function will be measured at different time points. The difference in responses in participants exposed and unexposed to DENV will be investigated.

1.7.2 Secondary endpoints

- Geometric mean JE neutralizing antibody titer (with 95% CI) and proportion of vaccinees with neutralizing antibody titers $\geq$ 1:10 30 days, and 1 year after vaccination in seroconverting vaccinees.
- Occurrence of adverse events after vaccination.
- Occurrence of serious adverse events for the entire study period in all participants.

1.8 Statistical Considerations

This is a descriptive study and no statistical comparisons are planned. Seroconversion will be analysed as geometric mean neutralisation titre and an interim analysis of safety data will be performed.

2. BACKGROUND AND INTRODUCTION

2.1 Japanese encephalitis

Japanese Encephalitis (JE) is a condition which affects primarily children and causes an acute febrile encephalopathy characterised by fever and clouding of consciouness, seizures, or other neurological symptoms. JE is endemic to South and Southeast Asia where JE epidemics occur regularly every year. JE is caused by an arthorpod-borne virus (arbovirus) called JE virus (JEV). The natural reservoir for JEV is wild birds and, in addition to the clinical JE seen in South and Southeast Asia, zoonotic JEV transmission is known to occur in subtropical regions of Asia like Japan and Korea. JEV is also spreading into Northern Australia. An estimated population of 30 million are at risk of getting JEV infection. There are approximately 50,000 sporadic and epidemic cases of JE every year. The ratio of clinical to subclinical infections has been estimated to be 1:270. Even if the clinical to subclinical ratio is taken as 1:100, annually nearly five million individuals get JEV infection. The mortality rate of clinical JE is between 25% to 40% and 60% to 70 % of the survivors have very severe neurological sequelae.

2.2 Epidemiology of JE in India

In India nearly all states have reported JE cases except that of Jammu and Kashmir and Himachal Pradesh. Acute febrile encephalopathy in children in India, however, has many causes and the causative agent often goes undetected. Over the years, JEV has been isolated from humans, pigs and mosquito vectors in India. In an endemic area, typically children between 5-15 years of age are affected. Annual peaks of incidence are seen associated with rainfall. In an area where JEV has been recently introduced adult cases are also reported. Many serological surveys in India have identified that JEV activity persists in many areas. The studies of recent infections in 0-15 age group indicated that JEV and West Nile virus (WNV) were equally active and dengue virus (DENV) was significantly less active. A serological survey carried out in the South Arcot district of Tamil Nadu in April 1982 showed a high prevalence of past JEV (49.17%), WNV (40.78%) and DENV (18.14%) infection with predominance of JEV. The JEV sero-prevalence did not differ between the villages with or without clinical JE.

2.2.1 Vectors and non-human hosts of JE virus

JEV is transmitted by Culex mosquitoes. In South India *Culex vishnui* and *C.tritaeniorhynchus,* have been identified as important for the spread and maintenance of JEV. JEV is maintained in nature in bird-mosquito-bird and pig-mosquito-pig cycles. JEV is also transmitted to the first generation progeny of the *Culex* mosquito vectors. Humans do not develop sufficient JEV viraemia for onward transmission and thus are dead end hosts in

the cycle of JEV in nature. The major reservoir of JEV in the environment is wild birds. JEV infected pigs develop viraemia but do not show sickness and therefore act as amplifying hosts for onward transmission to people because of their close proximity to human settlements.

2.3 Human immune responses to JEV

JEV is a single stranded, positive sense, enveloped RNA virus of the genus *Flavivirus*. The 11kb genome codes for three structural proteins: envelope glycoprotein (Egp), premembrane/membrane (prM/M) and capsid (C); and seven non-structural (NS) proteins denoted NS1, NS2a, NS2b, NS3, NS4a, NS4b and NS5. Given that, even in the absence of vaccination, the majority of people are protected against JE by their own immune response, differences in the immune response to JEV becomes a leading candidate for explaining why only some individuals develop JE after exposure to JEV.

2.3.1 Antibody responses

The dominant immune response in JE virus infection is against Egp, NS-1 and NS-3 proteins, where both antibody and T cell responses have been described. Traditionally, haemaglutinin inhibition (HI) and complement fixation (CF) tests have been utilized for characterization of flaviviruses along with neutralization tests. The HI response is essentially against Egp while CF antibody response is directed at both Egp and NS-1 proteins. It is known that immune response against Egp is virus neutralizing and protective and thus constitutes the major immune response in terms of vaccine development; there is also evidence of intrathecal synthesis of IgM in JE patients.

2.3.2 T cell responses

There are comparatively few studies on the cellular immune responses and interactions of JE virus with cells. JE virus multiplies in monocytes and activated T lymphocytes. Monocytes appear to be major target of the primary multiplication of JEV. IFNγ did not have any antiviral effect on JE virus multiplication. Although relatively little is known about the contribution of T cells to immunity to JEV T cell responses can be detected both in humans who have been asymptomatically infected and in JE cases. T cell proliferative responses during acute JE are rather weak but have been found to be more robust after recovery from infection. However, T cell IFNγ production to NS3 of JEV (a major T cell antigen) was poor in the same convalescent patients and there was an inverse correlation between IFNγ production and the degree of residual disability; in other words implying that there is a failure of the T cell response in those who develop overt JE. In recent years much more detailed studies of cellular immune responses against many other flaviviruses have been done, but JEV has lagged behind.

A major difference between live and killed vaccines is that live vaccines generally elicit stronger cell mediated immune responses. This in turn may lead to a longer degree of immune protection. For example the yellow fever virus (YFV) vaccine (in the same genus as JEV), one of the most successful vaccines ever made, can give detectable neutralising antibody 35 – 40 years after a single dose. It is known that YFV vaccine strain also elicits strong, broad, multifunctional and long lasting T cell responses. Live JE vaccine SA14-14-2 is known to elict T cell responses in mice (see section 2.7.1 below). There is no information, however, about human T cell responses to JEV SA14-14-2.

2.4 Vaccines against JE

2.4.1 Brief history of JE vaccines

Since the 1960s, both live and inactivated vaccines have been developed that provide active immunity against JEV. The development of these vaccines represents a major advance in the ability to control JE virus infection and reduce the burden of disease. Viruses isolated from human patients in Japan in 1935 and in China in 1949 provided the prototype Nakayama and Beijing and P3 strains, respectively, that are in principal use in the production of inactivated JE vaccine today. National vaccination programs in China (Province of Taiwan), Japan and the Republic of Korea, using an inactivated mouse-brain-derived vaccine that meets international requirements, have controlled the disease to the point of elimination, but in other countries, the expense and complexity of producing the vaccine and the need for repeated doses have limited vaccine use.

In addition to the problems posed by multiple doses, use of the vaccine to protect travellers has led to adverse events including hypersensitivity and demyelinating disease. Adverse events have been reported in North America, Australia and Europe. The live-attenuated JE vaccine SA14-14-2 (PHK strain) was developed in China as an alternative to inactivated vaccines. Since its licensure in China in 1988, more than 300 million doses of live JE vaccine have been produced for administration to children in annual vaccination programs. The vaccine is of considerable interest to countries where JE virus is endemic and is also licensed in several other countries in Asia.

2.4.2 Mouse brain derived killed purified vaccine

The existing vaccine for JE is purified, killed virus vaccine prepared from infant mouse brain that consists of mainly Egp of the virus. To be effective three doses of the injectable vaccine are to be administered. Mouse brain vaccine has been produced in India at the Central Research Institute Kasauli and it also elicits neutralizing antibodies against Indian strains of JEV.

Various trials with the mouse brain inactivated vaccine have been carried out, with efficacy noted only if three doses are administered. For example, a trial with vaccine made in Japan was carried out in South Arcot district of Tamil Nadu, India. Of a total of 113 school children, 72% showed an antibody response increasing to 87.8% after a booster dose of Biken JE vaccine at one year. Only about 20 per cent of the children had persisting antibodies one year after the primary vaccination.

2.4.3 Live attenuated JE vaccine

Attenuated virus vaccines have always been preferred over the killed vaccines as they mimic the exact grade of immune response that is required for protection from the wild type. The SA14-14-2 strain was derived in China in the 1980s by multiple passages (see below) and has been widely used in China since 1988. The vaccine has been licensed in Nepal in 1998, in South Korea in 2001, in Sri Lanka in 2003, in India in 2006 and in Thailand in 2007. Many hundreds of millions of doses have been used throughout Asia. The live JE vaccine is not yet pre-qualified by the WHO, though work towards this is at an advanced stage of progress. A new production facility to manufacture live JE vaccine to international good manufacturing practice (GMP) standards is due to commence production in China in early 2011.

2.5 Brief description of live JE vaccine SA14-14-2

2.5.1 Vaccine

The live JE vaccine is a preparation of live attenuated JEV strain SA14-14-2 grown on a mono-layer of primary hamster kidney cell cultures. After cultivation and harvest an appropriate stabilizer is added in the virus suspension, which is then lyophilized. Live attenuated JEV SA14-14-2, MEM (Minimum Essential Medium), human serum albumin are the major components of the final vaccine. The vaccine is used to prevent JE.

The SA14-14-2 strain grows to a titre of $>10^7$ in PHK cells and produces a cytopathic effect and small plaques under overlay. The use of PHK cells for routine production of JE vaccine (live) is clearly effective, but raises particular cell substrate issues (below).

The live attenuated JE vaccine is resuspended in diluent (provided with the vaccine) immediately prior to use and administered via subcutaneous injection at the deltoid insertion area of the upper arm.

2.5.2 Derivation of the SA14-14-2 vaccine strain of JE virus

The wild-type parental virus, SA14, was isolated from a pool of *Culex pipiens* larvae from Xian, China. The derivation of the SA14-14-2 strain was through an empirical process of serial passage, principally in Primary Hamster Kidney (PHK) cells, and demonstrated that a

fine balance exists between safety through stable neuroattenuation and immunogenicity, with sufficient viral replication to stimulate immunity.

The parent virus (JEV SA14) was passaged 11 times in mouse brain, around 100 times in PHK cells and subject to three plaque purifications to generate the intermediate strain SA14-12-1-7. Six animal passages and six plaque purifications yielded strain SA14-5-3 which had a stable neuroattenuated phenotype but lacked sufficient immunogenicity. A further five animal passages and two plaque purifications gave rise to the final attenuated strain SA14-14-2 with adequate immunogenicity.

An initial attempt to adapt the SA14-14-2 strain to primary dog kidney cells found that only nine additional passages led to further attenuation and a reduction in its immunogenicity, yielding seroconversion in only 40% of vaccinated children.

2.5.3 Cell culture substrate

Prevention of transmission of adventitious infections from the virus seed, the cell substrate, and the serum or trypsin used in the manufacturing process, is a general concern with all live-virus vaccines. For the SA14-14-2 vaccine, The attenuation process was originally carried out in hamster kidney cells that have unknown passage histories. In this case the lack of precedence for a PHK cell substrate in live attenuated vaccine is a special issue. However, PHK is recognized as an acceptable substrate for inactivated JE vaccine and *hantavirus* vaccine (Haemorrhogic Fever with Renal Syndrome (HFRS) vaccine). Current controls cover a broad range of potential rodent virus contaminants.

The principle of reducing the risk of adventitious agents entering the manufacturing process is increased by using healthy animals, from a closed specific pathogen-free colony that is monitored regularly, as a source material for preparation of PHK cells. In common with all live-virus vaccines, steps to exclude potential contaminants of serum and trypsin employed in manufacturing, including specific bovine and porcine viruses and transmissible spongiform encephalopathy agents are performed by using materials of certified origin.

2.6 Characterisation of live JE vaccine SA14-14-2

Characterization and immunogenicity studies on SA14-14-2, its parent wild type strain SA14 and SA14-5-3 have been carried out. Wild-type strain SA14 was found to be a poor immunogen and antigenically distant from all other viruses examined. The vaccine derivatives SA14-5-3 and SA14-14-2 were more immunogenic than its wild-type parent and elicited a cross neutralizing antibody response. Hyperimmunization with either Nakayama strain elicited equally good neutralizing antibody response. Antigenic variation between wild-

type and vaccine clones of JE virus were detected, but were not considered significant in terms of controlling JE virus infections by vaccination.

2.6.1 Pathogenicity of SA 14-14-2

In contrast to its parent strain, the SA14-14-2 strain is avirulent when administered by the intracerebral (i.c.) and intraperitoneal (i.p.) routes in weanling mice, Syrian hamsters and in mice given immunosuppressive treatment with cytoxan. The virus is virulent for nu/nu mice only when administered by i.c. inoculation, but with a longer incubation period than the parent strain SA14.

2.6.2 Tests in monkeys

A freeze-dried primary/master seed lot with a titre at least 5.7 log PFU/ml was used to conduct neurovirulence tests in monkeys. The primary/master seed virus were diluted five fold (1:5) and given to ten rhesus monkeys by inoculation of 0.5 ml into the thalamic region of each hemisphere and 0.2ml into the lumbar sponal cord. For the control groups, the virulent strain (SA14) was diluted at 10^2 PFU/ml and 10^3 PFU/ml and administered to four rhesus monkeys each using an identical protocol.

The ten monkeys in the test group of attenuated virus SA14-14-2 were observed for at least 18 days and no clinical symptoms were observed. Only mild inflammatory reactions were found along the needle track at the injection sites of brain and spinal cord in histopathological examinations. In the control group, after 8 days, all of the four monkeys in 10^3 PFU/ml group and at least two of the four monkeys in 10^2 PFU/ml group died. Histopathological analysis of the monkeys in the control groups showed neuronal necrosis and inflammatory reactions.

2.6.3 Tests in mice

At least 10 mice weighing 12-14 g were injected i.c. with 0.03 ml of virus seed lots and observed for 14 days. Up to 10^6 infectious units of the SA14-14-2 strain produced no illness, indicating a high level of neuroattenuation. (Mice that died within 3 days after inoculation were considered to have died from the process of i.c. innoculation and were excluded from analysis; a valid test required that no more than 20% of mice to die within 3 days.) After 3 days, the mice showing symptoms were killed, and their brains were removed and tested for signs of neurovirulence. The i.c. LD_{50} titer did not exceed 3.0 log LD50/0.03ml. One infectious unit of the wild-type parent SA14 virus was uniformly fatal for weanling mice after i.c. inoculation.

Small plaque morphology and neuroattenuation in mice is retained through at least 23 further PHK cell passages, using conditions of infection (e.g. multiplicity of infection and incubation

temperature) identical to those employed in production. Mice were more sensitive than monkeys to i.c. infection, an observation that allowed mice to be used in release testing.

2.6.4 Test for reversion of neurovirulence in mice

Ten 3-5 day old suckling mice were injected i.c. with 0.02 ml of at least 7.2 log PFU/ml liquid virus seed or at least 5.7 log PFU/ml freeze dried virus seed. Three of the suckling mice showing symptoms of JE were killed and their brains were removed and emulsified. The i.c. LD_{50} titer did not exceed 3.0 log LD_{50}/0.03ml in each mice weighing 12-14 g. Then a second group of at least ten fresh mice weighing 10-12 g were inoculated s.c. with 0.1 ml of 10^{-1} brain suspension from these sick mice showing symptoms of JE after SA14-14-2 i.c. infection. No mice in the second group showed symptoms of JE viral infection during the 14-day observation period, indicating that the virus recovered from mice showing symptoms after i.c. infection had not reverted to a virulent phenotype.

2.6.5 Molecular basis of attenuation

In order to understand the molecular basis of attenuation, SA14 parent virus and the attenuated SA14-14-2 virus have been sequenced and nucleotide sequences studied. Nucleotide substitutions were found to be scattered all over the genome. Of these, 24 resulted in amino acid changes within viral proteins. Structural proteins C and Egp contain one and eight amino acid changes, respectively. Of the nonstructural proteins, NS1 contains three, NS2a two, NS2b-two, NS3-four, NS4a-one, NS4b-one, and NS5-two amino acid substitutions. Mutations observed were in the Egp at 138, 176, 315 and 439, while in NS2B 63, NS3 105, and NS4B 106 amino acid positions. The mutations in NS2B and NS3 are in functional domains of the trypsin-like serine protease. Although these changes are described the precise molecular reasons for attenuation remain unknown, due in part to the absence of a cDNA clone of JEV to allow investigation of the role of specific molecular changes by site directed mutagenesis.

2.6.6 Potential for mosquito-borne transmission of the vaccine virus

Although the growth of SA14-14-2 virus in *Culex tritaeniorhynchus* has not so far been evaluated, the attenuated SA14-1-8 clone, derived from the same pedigree with a similar phenotype, showed no transmission in experimental studies. Among 60 pools of *C. tritaeniorhyncus* and *C. pipiens* mosquitoes fed on a 106.2 PFU/ml inoculum in oral pledgets, virus at a low level (<101.5 PFU/ml) was detected in two pools, 5 to 6 days after feeding. Experimental transmission of the SA14-14-2 strain by vector mosquitoes is under study. In contrast, the SA14 parent was transmitted in mosquitoes at rates of 75–78%. Recent studies to assess the extent of detectable viraemia following vaccination with the SA14-14-2 strain showed that JEV SA14-14-2 viraema is undetectable following immunisation in humans.

These results are consistent with previous reports showing that wild-type JEV is undetectable or rarely detectable in infected symptomatic persons and that there is no amplification of JEV in humans. In view of the above, and the estimated requirement for 10^5 to 10^6 virions per ml of plasma for mosquitoes to access sufficient virus from a human to transmit to others, it is extremely unlikely that the SA14-14-2 strain would be transmitted via a mosquito bite of a recent vaccinee.

2.7 Specific Pharmacological Actions

2.7.1 Efficacy (immunogenicity and challenge protection) studies in animals

In vaccination/challenge studies, mouse survival was significantly greater after vaccination with one dose of live vaccine compared with two doses of inactivated vaccine (derived from mouse brain or PHK cells), followed by i.p. challenge with the P3, Nakayama or 12 field strains isolated in China (survival 90% versus 60% after one month; 100% versus 33% after six months, table 2.7.1). Protection was also greater after i.c. challenge (table 2.7.2). In addition to a higher level of protection SA14-14-2 live vaccine induced a longer duration of immunity in mice Compared with two doses of killed PHK vaccine.

Table 2.7.1: Comparison of protective effect and duration induced by live and killed vaccines

Vaccines	Protection rates (% survival)		
	1m	3m	6m
Live SA14-14-2 (1dose)	90% (9/10)	87.5% (7/8)	100% (9/9)
Killed PHK (2 doses)	60% (6/10)	50% (5/10)	33% (3/9)
Control	0% (0/10)	10% (1/10)	0% (0/9)
Challenge virus (Log_{10} i.p. LD50/0.3ml)	3.17	4.58	3.77

Table 2.7.2: Comparison of protective effect in mice by intracerebral challenge between live and mouse brain killed vaccines

Vaccine	Nab (Pre-challenge)		Protection*			
			P3		Nak	
	P3	Nak	ic	ip	ic	ip
Live SA14-14-2(1x)	20	40	8/10	10/10	4/10	10/10
MBV Killed(2x)	20	40	3/10	8/10	1/10	4/10
Challenge virus dose ($\log_{10}$ LD_{50}/0.03ml)			1 .0	4.7	2.0	7.3

ic, intracerebral challenge; ip, intraperitoneal challenge

*: No. of survivors/No. tested.

Nab = neutralising antibody

Evidence that the live vaccine elicited a stronger cellular immune response than inactivated vaccine was seen in these experiments; despite equal titers of circulating neutralizing antibody, survival after i.c. challenge was significantly higher in animals previously immunized with live vaccine than in those immunized with inactivated vaccine (80% versus 30%). Cytoxan immunosuppression of mice vaccinated with SA14-14-2 did not alter their resistance to lethal viral challenge in contrast to mice vaccinated with inactivated vaccine, in which survival was reduced by 90% after immunosuppression. Mice vaccinated with live vaccine developed demonstrable cytotoxic T cell activity (79.2%), while none was detected in animals inoculated with PHK killed vaccine (activity level similar to control animals, table 2.7.3).

Table 2.7.3: Comparison of CTL activities induced by different vaccines

Vaccine	Cytotoxicity CTL %
Live SA14-14-2 (1 dose)	79.2 ± 1.08*
Killed PHK (2 doses)	29 ± 3.04
Control	24.6 ± 1.35

* mean ± SD from 3 tests

The contibution of cellular immunity was demonstrated by adoptive transfer of immune spleen cells from mice immunized with live vaccine to native mice. Survival was 50% after challenge with wild type virus; whereas, no protection was seen when immune spleen cells were transferred from mice immunized with killed vaccine (10% survival, similar to the rate in control mice).

Table 2.7.4: Comparison of protective effect of adoptive transfer of immune spleen cells induced by different vaccines in recipient mice**

Immune Vaccines	Protection*
Live SA14-14-2 (1x)	5/10 (50%)
Killed (PHK) (2x)	1/10 (10%)
Control	1/10 (10%)

***No. survivors/ No. tested**

**Recipient mice received spleen cells and challenged i.p. with 4.0 log LD50 virus.

Animals vaccinated with live JE vaccine in which neutralizing antibody titers had waned developed an anamnestic neutralizing antibody response to viral challenge that was protective.

Guinea-pigs immunized with one dose of live JEV vaccine SA14-14-2 and challenged i.p. 30 days later with wild-type JE virus developed neutralizing antibodies rapidly, from a titer <2–8 in pre-challenge serum samples to 8–32 on day 4 and 128–256 on day 5 post-challenge. In contrast, neutralizing antibodies rose slowly in the control animals and were not detectable until day 7, with low titers of 2–4. Viremia was totally suppressed in vaccinated guinea pigs after challenge; whereas, in all control animals viremia with high titers (2.0–3.54 logs) circulated on day 2 post-challenge, lasting 4 days. Either spleen cells or serum from vaccinated animals could passively transfer immunity to un vaccinated animals.

2.7.2 Heterogenotypic coverage

JE live vaccine exhibited protective efficacy against a broad spectrum of JE viral genotypes. A single dose of the live vaccine protected against lethal challenge with JE viral strains isolated from different locations in China and with field strains isolated in India, Indonesia, Japan, the Philippines, Thailand and Viet Nam. Protection rates reached 90% to 100% when 340 PFU/0.1ml of the vaccine virus was used as the immunizing dose, and even vaccine

inocula as low as 34 PFU virus protected 70% to 100% of challenged mice. The live vaccine therefore provided a high level of protection against a broad spectrum of JE viral genotypes.

2.7.3 Animal toxicology

No data have been generated regarding non-clinical toxicology as the live JE vaccine SA14-14-2 was developed in the 60s. However, live attenuated JE vaccine, like any live attenuated vaccine, potentially carries an additional risk on pregnant women and immunocompromised patients.

Although experimental data suggest that live JE vaccine SA14-14-2 is not neurotropic in immunosuppressed animals, there are no data on the vaccine's safety in immunocompromised individuals, for example HIV-infected patients.

2.8 Summary of clinical safety studies

SA14-14-2 live JEV vaccine was licensed in China in 1988 and since then around 20 million doses per year have been used with little evidence of serious adverse events. Although few rigorously conducted studies have formally assessed the safety of SA14-14-2, wide experience of its use in many countries in Asia makes the occurrence of any serious risk very unlikely. In one large clinical trial in China a wide range of mild and moderate adverse events were reported but serious adverse events (seizures, hospitalisation) were not common in the vaccinated subjects than in control subjects. Since then other trials have been conducted in the Phillipines, Sri Lanka and India. Without exception the safety profile of JE vaccine SA14-14-2 has been excellent in these more recent trials, none of which have reported any serious adverse events. It should be noted that the safety of this live vaccine in the immunocompromised has not been assessed. Case control studies indicate that the vaccine efficacy is 80-99% depending in part upon the incidence of JE in the trial locality.

SA14-14-2 is licensed for use in India where millions of doses have been delivered since 2006. At the beginning of the campaign in 2006 there were 9.3 million doses of SA14-14-2 delivered with 65 serious adverse events and 22 deaths reported; however upon investigation there was no clear link with SA14-14-2 vaccination. Recent studies have also showed good efficacy; generally the endpoint measured is the plaque reduction neutralisation titre (PRNT) against JEV SA14-14-2 at various time points following vaccination (typically one month, six months and one year).

2.9 Rationale for study

In natural infection with Flaviviruses (the family of viruses to which JEV belongs) T cell responses are readily detetctable. In recent years great progress has been made in understanding T cell responses to a number of Flaviviruses particularly West Nile virus

(WNV) and dengue virus (DENV). In addition two detailed studies have described the immune response, including the T cell response, to yellow fever vaccine. In humans, the function of these T cell responses remains unclear though in animal models T cell responses can be shown to be involved in anti-Flavivirus immunity. JE is arguably one of the most important Flaviviruses to infect humans in terms of morbidity and mortality, yet our knowledge of the T cell response to JEV has fallen behind that of other Flaviviruses. For example, there are currently no optimal human T cell epitopes defined for JEV in the literature. Studying the T cell response in a controlled environment, such as after vaccination, offers an opportunity to develop a far greater understanding of the normal T cell response to JEV in a setting where disease does not develop. This knowledge can be used as a basis to build an understanding of the differences that occur in immune responses in acute disease, as well as providing knowledge on the basic function of the immune system after this vaccine such as the duration of protection.

Flaviviruses are highly immunologically cross-reactive. However, whether or not this cross-reactivity can contribute significantly to protection is unknown. This is a particular problem in dengue virus infection where cross-reactivity also contributes to the development of more severe disease. As work on dengue vaccines progresses cellular immune outcomes after vaccination are increasingly being studied. A detailed study on live JE vaccine SA14-14-2 to identify the major T cell epitopes and investigate cross-reacting epitopes with other flaviviruses will provide useful information to help avoid confusion in other vaccine studies in areas where DENV and JEV co-circulate. This study will also investigate whether there is cross-priming between DENV and JEV that affects the susbsequent response to JE vaccination. Prior DENV infection might have a positive, a neagtive, or no effect on the immune response to JE vaccination. Interestingly, however, this might clarify why a single dose of live JE vaccine SA14-14-2 has been noted to be more effective in India (mainly a DENV endemic area) than in China. This is despite evidence from a JE vaccine trial in Sri Lanka that DENV infection before the final dose of live JE vaccine SA14-14-2 in fact *reduced* the anti-JEV neutralisation titre (though not below the level of protection). This suggests that perhaps the difference is accounted for by the T cell response.

3 METHODS

3.1 Study Design

Prospective, open-label, uncontrolled study in JEV/WNV seronegative participants administered a single dose of SA14-14-2 vaccine.

3.1.1 Trial sites:

- Indian Institute of Science, Bangalore 560012
- Dept. of Neurovirology, NIMHANS, Bangalore 560029

Information sheets and advertisements would be distributed and put in the above centers after approval from the ethics committee.

3.1.2 Trial period:

May 2012 – May 2013

3.1.3 Study population:

Healthy adults aged 18 – 50 years.

3.1.4 Objectives:

3.1.4.1 Primary objective

- To describe the nature, quality and duration of the human T cell response to live attenuated Japanese encephalitis (JE) vaccine SA14-14-2

3.1.4.2 Secondary objectives

- To determine seroconversion to the vaccine at one month, six months and one year after vaccination
- To correlate the quantity and quality of the T cell response with the antibody response.
- Occurrence of adverse events after vaccination.

3.1.5 Number of participants planned

Total number of participants:	20
Expected drop out rate:	25%
Approximate number to be enrolled:	25
Expected screen failures:	80%

Prospective participants will be screened until a sufficient number of eligible participants are identified. We aim to have approximately equal numbers of dengue virus (DENV) exposed and unexposed individuals.

3.1.6 Inclusion criteria:

- A male or female adult between 18 and 50 years of age.
- Written informed consent.
- Free of obvious health problems as established by medical history and history-directed physical examination before entering the study.
- Expected continuous residence in India during study period, without travel outside India.
- An efficacious method of contraception must be used during the study phase for women of child bearing age.

3.1.7 Exclusion criteria:

- Use of any investigational or non-registered drug or vaccine other than the study vaccine within 30 days preceding administration of SA14-14-2 vaccine, or planned use during the study period.
- Chronic administration (defined as more than 14 days) of immunosuppressants or other immune-modifying drugs within a period of six months before vaccination or at any time during the study period. (For corticosteroids, this means prednisone, or the equivalent, ≥0.5 mg/kg/day. Inhaled and topical steroids are allowed.)
- Any confirmed or suspected immunosuppressive or immunodeficient condition.
- A family history of congenital or hereditary immunodeficiency.
- Any antiviral drug therapy within a period of six months before vaccination or at any time during the study period.
- History of significant allergic disease (e.g., anaphylaxis to foods, drugs, vaccines or hymenoptera) or reactions likely to be exacerbated by any component of the study vaccine, especially allergic disease or reactions to any previous dose of any vaccine.
- History of having received JE vaccine, yellow fever vaccine, tick-borne encephalitis vaccine or experimental flavivirus vaccine.
- History of documented JE infection.
- Detectable anti JE neutralizing antibodies in screening tests.

- Acute disease at the time of enrollment. Entry into the study may be deferred until the illness is resolved. (Acute disease is defined as the presence of moderate or severe illness with or without fever. All vaccines can be administered to participants with a minor illness such as diarrhea or mild upper respiratory tract infection with or without low-grade febrile illness, i.e., temperature <37.5°C.)
- Acute or chronic, clinically significant pulmonary, cardiovascular, hepatic, neurological or renal functional abnormality, as determined by history and physical or laboratory examination that is not controlled by drugs.
- Administration of immunoglobulins and/or any blood products within the three months preceding administration of vaccine, or planned administration during the study period.
- Seropositive for HIV, HCV or HbsAg.
- Lactation, pregnancy or intention to get pregnant.
- History of excessive alcohol consumption, drug abuse or significant psychiatric illness.
- Any other condition that in the opinion of the investigator would pose a health risk to the participant or interfere with the evaluation of the vaccine.

3.1.8 Endpoints

3.1.8.1 Primary endpoint

The primary endpoint of this study is a description of the T cell immune response to the live attenuated JE vaccine SA14-14-2. Peripheral blood mononuclear cells (PBMC), which include peripheral blood T cells, will be stimulated *in vitro* using a synthetic peptide library covering the entire genome of the virus. This will allow the determination of where within the virus the important T cell antigens reside, according to HLA type. Markers of activation, proliferation, apoptosis and immune function will be measured at different time points. The difference in responses in participants exposed and unexposed to DENV will be investigated.

3.1.8.2 Secondary endpoints

- Geometric mean JE neutralizing antibody titer (with 95% CI) and proportion of vaccinees with neutralizing antibody titers $\geq$ 1:10 30 days, and 1 year after vaccination in seroconverting vaccinees.
- Occurrence of adverse events after vaccination in all participants.
- Occurrence of serious adverse events for the entire study period after vaccination in all participants.

3.1.9 Statistical Considerations

This is a descriptive study and no statistical comparisons are planned. Seroconversion will be analysed as geometric mean neutralisation titre and an interim analysis of safety data will be performed.

3.1.9.1 Statistical Analysis

The analysis will be descriptive. T cell responses will be quantified in terms of number of interferon-gamma (IFNγ) spot forming cells/million PBMC and the median and interquartile range reported; and by descriptive analysis of the identified epitopes. The proportion of cells responding to individual epitopes will be quantified as a percentage of CD8+ T cells using HLA/peptide tetramers or cytokine production. Polyfunctional/cytokine secreting cells and activation markers will be expresed as percentages of relevant T cell substes (CD4+, CD8+ etc). Neutralizing antibody response will be reported as percentages of achieving participants antibody titers in twofold dilutions beginning at 1:10. The GMT will also be reported with 95% CI.

3.1.9.2 Interim Analysis

An interim analysis of data pertaining to adverse events (AE) obtained after the vaccination for all participants will be compiled. The AEs will be tabulated to display severity and intensity of AEs. The final analysis of safety data will be performed at the end of study.

3.2 Screening and visits

3.2.1 Baseline Screening: Potential participants will be screened for JEV and WNV antibodies, JE T cell response by ELISPOT. Blood will be tested for HIV, HBsAg, HCV, haematology (haemoglobin, white cell count, platelet count) and biochemistry (Urea, creatinine, electrolyte and glucose). Urine will be collected for urinalysis. A standard medical history and physical examination including vital signs will be performed. Urine pregnancy test will be performed in women of child bearing age.

3.2.3 Pre vaccination: The participant's interim medical history will be obtained. A physical examination including vital signs will be performed. Urine pregnancy test will be done in women of child bearing age. Blood will be drawn for testing. The sample will include 10ml for serology assays, 2.5 ml for DNA (for HLA typing), 2.5 ml for RNA isolation, which must be done before vaccination.

3.2.3 Visit 1 (Day 0): After the participant's eligibility has been confirmed, the participants will be vaccinated. The participants will then be observed by the study doctor at the vaccination facility for 30 minutes in order to document and treat any acute reactions, including

anaphylaxis. Because it is necessary to know the ELISPOT assay result before vaccination, the pre-vaccination visit must be at least two days before the vaccination visit, and should not be more than 2 weeks before the vaccination visit.

3.2.4 Visit 2 (Day 7): Interim medical history will be obtained. Physical examination including vital signs will be performed if clinically indicated. Blood will be drawn for PBMC separation, RNA isolation and serum.

3.2.5 Visit 3 (Day 14) and subsequent visits: Interim medical history will be obtained. Physical examination including vital signs would be performed. Blood will be drawn for PBMC every two weeks for 12 weeks, every month from 4th month to 6th month and every 2 months from 6th month to one year. Pregnancy test will be done every two months for the entire study period. Further blood test will be done based on clinical indication. The full visit schedule is shown in table 3.2.5

Table 3.2.5 Schedule of Events

Visit	Serum Analysis	PBMC Separation	RNA isolation	DNA HLA typing	Haematology/ biochemistry/ Urinanalysis	Pregnancy test
Pre vaccination	✓	✓	✓	✓	✓	✓
7th day	✓	✓	✓			
14th day	✓	✓				
28th day	✓	✓				
6th week		✓				
8th week		✓	✓			✓
10th week		✓				
12th week	✓	✓				
4th month to 6th month, every month	✓	✓				✓ (every two months)

6th month to 18th month, every two months	✓	✓				✓

3.3 Concomitant Medications

Participants may take all other medications as clinically indicated. However, if he/she receives immunosuppressive medication, the participant will be discontinued from the study sampling schedule. Safety-related follow-up will be continued for the length of the study. All medications whether prescription or non-prescription during the study as well as any medications used in the treatment of an adverse event will be recorded on the concomitant page of the CRF.

3.4 Procedure for monitoring compliance

If the participants do not turn up on the scheduled date, repeated attempts to contact the participant will be made, after which the participant would be deemed as lost to follow up.

4. SUMMARY OF STUDY REQUIREMENTS

4.1 Investigational material

Live attenuated JE Vaccine is licensed and widely used in India. It is a preparation of live attenuated JEV (strain SA 14-14-2) grown on a primary hamster kidney (PHK) cell monolayer. After cultivation and harvest an appropriate stabilizer is added in the virus suspension, which is then lyophilized. Live attenuated JE virus (strain SA 14-14-2), MEM (Minimum Essential Medium), human serum albumin are the major components of the final vaccine. The vaccine is used to prevent JE. The vaccine will be shipped under monitored refrigerated conditions.

Table 4.1.1 Composition of lyophilized JE Vaccine, Live in the 1-dose vial

INGREDIENT	QUANTITY	FUNCTION
Active Substance		
Live attenuated JEV strain SA 14-14-2	≥ 5.4 log PFU	Immunizing antigen
Excipients		
Gelatin	4.8 mg	Stabilizer
Sucrose	21 mg	Stabilizer
Lactose	21 mg	Stabilizer
Carbamide	2.4 mg	Stabilizer
Human serum albumin	≤ 3 mg	Stabilizer

4.2 Reconstitution and Administration of the Vaccine

Single dose lyophilizate should be reconstituted with 0.5 ml of water for injection (WFI) and 5-dose lyophilizate with 2.5 ml of PBS. After reconstitution, it turns into a transparent orange red or light pink liquid. Each single dose should not contain less than 5.4 log PFU of live JEV SA14-14-2. It will be administered via subcutaneous injection at the deltoid insertion area of the upper arm. The reconstituted vaccine is stable at room temperature for 6 hours and hence must be used within 6 hours.

4.3 Vaccine Accountability

Dedicated vaccine accountability records will be kept. The vaccine will be stored according to the manufacturer's recommendations at 2°C to 8°C in a refrigerator, the temperature of which will be monitored. Written records of temperature, receipt and storage of the vaccine will be kept. This will include: date received, lot number, quantity received, state in which received, temperature of vaccine, and dose administered, with the coded identification of the participant, and disposal or return to the sponsor/authorized distributor. Any known discrepancies in the accountability of the vaccine will be documented. The investigator will not use the vaccine in any other manner than that provided for in the protocol. Additional vaccine vials may be provided to replace broken or lost doses. All used, unused and broken vials will be retained for accountability purposes and may be destroyed as per manufacturer's instruction and WHO guidelines. All reconstituted vaccine not used within 6 hours after reconstitution during an immunization session will be discarded as per normal practice.

5. ASSESMENT OF SAFETY

5.1 Safety Monitoring

If any of the following have occurred and are deemed by the Investigator to be probably or possibly related to the vaccine, the trial will be interrupted (it should be stressed that with millions of doses of vaccine given in India there have been no such events conclusively linked to the vaccine):

- Any of the following severe reactions in one or more participants: pneumonia or encephalitis/ encephalopathy.
- Axillary temperature >39.5°C in more than 3 participants within 14 days after vaccination.
- A serious adverse event except anaphylaxis in one or more participants.

5.2 Discontinuation criteria/Stopping rules

Participitants will be discontinued from the study, or the trial will be discontinued (as appropriate) if-

- If a participant becomes pregnant, all data for previous month will be disregarded and data upto the last negative pregnancy test will be used. The participant will leave the study sampling schedule, but safety follow up will continue until the end of study.
- If there is a life threatening event possibly related to vaccine or any other reason at the discretion of the PI, safety monitor or IHEC.

5.3. Adverse Events

An **adverse event** (AE) is any untoward medical occurrence in a clinical trial participant to whom a vaccine has been administered; it does not necessarily have a causal relationship with the vaccine/vaccination.

An **unexpected adverse event** is one whose nature or severity is not consistent with the applicable product information (e.g. vaccine product information sheet).

A **Serious Adverse Event** (SAE) is any adverse event that:

1) results in death;
2) is life threatening;
3) requires or prolongs hospitalization;
4) results in persistent or significant disability or incapacity;
5) results in a congenital anomaly/birth defect or malignancy.

(It should be stressed that with millions of doses of vaccine given in India there have been no such events conclusively linked to the vaccine, and such events are not anticipated to occur during the study.)

Categories	Adverse Event	Grade 1	Grade 2	Grade 3	Grade 4
Local Reaction	Pain	Does not interfere with activity	Interferes with activity or repeated use of non-narcotic pain reliever	Prevents daily activity or repeated use of narcotic pain reliever	Emergency room (ER) visit or hospitalization
	Erythema/ Redness*	2.5 - 5 cm	5.1 - 10 cm	> 10 cm	Necrosis or exfoliative dermatitis
	Swelling **	2.5 - 5 cm and does not interfere with activity	5.1 - 10 cm or interferes with activity	> 10 cm or prevents daily activity	Necrosis
General (systemic) AEs	Fever (°C)<br>(°F)	38.0 - 38.4<br>100.4 - 101.1	38.5 - 38.9<br>101.2 - 102.0	39.0 – 40<br>102.1 - 104	>40<br>> 104
	Nausea/ vomiting	No interference with activity or 1 - 2 episodes/24 hours	Some interference with activity or > 2 episodes/24 hours	Prevents daily activity, requires outpatient IV hydration	ER visit or hospitalization for hypotensive shock
	Loss of appetite	No interference with activity	Some interference with activity not requiring medical intervention	Prevents daily activity and requires medical intervention	ER visit or hospitalization

Table 5.3 Anticipated Adverse Events

** Swelling should be evaluated and graded using the functional scale as well as the actual measurement. In addition to grading the measured local reaction at the greatest single diameter, the measurement should be recorded as a continuous variable.

5.4 Assessment of Causality

The investigator will assess whether the reaction is related to the immunization using the following scale and according to the following definitions (based on World Health Organization, Adverse Events Following Immunization: Causality Assessment):

- **Very Likely/Certain:** A clinical event with a plausible time relationship to vaccine administration and which cannot be explained by concurrent disease or other drugs or chemicals.
- **Probable:** A clinical event with a reasonable time relationship to vaccine administration; is unlikely to be attributed to concurrent disease or other drugs or chemicals.
- **Possible:** A clinical event with a reasonable time relationship to vaccine administration, but which could also be explained by concurrent disease or other drugs or chemicals.
- **Unlikely:** A clinical event whose time relationship to vaccine administration makes a causal connection improbable, but which could be plausibly explained by underlying disease or other drugs or chemicals.
- **Unrelated:** A clinical event with an incompatible time relationship and which could be explained by underlying disease or other drugs or chemicals.
- **Unclassifiable:** A clinical event with insufficient information to permit assessment and identification of the cause.

In the unlikely occurance of any adverse events the participants will be followed up until a stable clinical endpoint is achieved.

5.5 Treatment of Adverse Events

Treatment of any AE related to the vaccine will be conducted by the Investigators and should be in accord with accepted, local standards of good medical practice. Participants will not be charged. The participants will be instructed to contact the Investigators immediately should they manifest any sign or symptom perceived as serious.

5.6 Adverse Event Follow-up & Assessment of Outcome

Investigators should follow participants with AEs until the event has subsided, or until the condition has stabilized. Any adverse events will be recorded. All adverse events report will be reported to the PIs, Safety Monitor and IHEC. In the extremely unlikely event of any serious adverse event, the event will be reported by telephone or fax to the local safety monitor within 72 hrs of identification of their occurrence. Within 5 working days, a complete report would be completed and sent to the local safety monitor. If any additional information regarding this event becomes available, it will be forwarded to the local safety monitor, PI and IHEC as soon as possible.

Contact detailes of the local safety monitor:

Dr Nagabhushana

Chief Medical Officer

Indian Institute of Science

Bangalore 560029

Ph no: 080 2293 2552

Email id: nag@admin.iisc.ernet.in

5.7 Pregnancy

Participants cannot become pregnant during the course of the study. Participitants who become pregnant during the study period will be discontinued from the study sampling schedule. The data collected up untill the last prergnancy test will still be used.

The pregnancy will be followed to term, any premature terminations will be reported, health status of the mother and child including date of delivery, child's gender and weight should be reported to the sponsor. The investigator should document the progress of the child's development until 12 months of age.

6. LABORATORY

All blood tests (except serology) will be performed at RV laboratories, Malleshwaram, Bangalore (supplier of lab services for the IISc health centre) or NIMHANS.

6.1 Haematology/Biochemistry/Urinalysis

Hematology profile will comprise hemoglobin, white blood cell count and platelet count. Biochemistry profile will comprise glucose, sodium, potassium, creatinine. Urinalysis will comprise colour, pH, specific gravity, protein, glucose, white blood cells and red blood cells.

6.2 Serology

Screening serologies for JEV and WNV will be done by ELISA (InBios, International Inc, Seattle, USA). JEV antibody titres will be determined by a plaque reduction neutralization test (PRNT).

6.3 Specimen handling and analysis

Blood will be transported in a timely manner. Serum specimens will be aliquoted into 3 samples and stored at $-70^{o}C$ at Indian Institute of science. PBMC will be separated immediately (within hours) for all participants except for occasions when experiments are done on whole blood. Wherever possible T cell assays will be done immediately on fresh cells. Where this is not possible cells will be crypreserved using standard protocols are revived and rested overnight prior to use.

6.3.1 Archiving and tracking of samples

Samples will be aliquoted, processed and stored at Prof Vijaya's laboratory at IISc, according to Indian guidelines. The mechanical functioning of freezers is monitored by alarms and back-up systems. A secure database at IISc will be used for tracking and monitoring the submission, receipt and location of all specimens ensuring efficient retrieval of samples. Confidentiality of participants will be maintained by storing samples using a coded identifier that links the sample to the clinical data.

6.4 Immunological experiments

Participants' heparinised blood samples will be used for peripheral blood mononuclear cell (PBMC) separation. PBMC will be cryopreserved when there are too many samples to perform all the experiments at one time. On occasion stimulations or staining for flow cytometry will be done directly on whole blood.

PBMC will be screened against pools of peptides derived from a commercial peptide library of JEV SA14-14-2. Responses will be assayed by interferon-gamma (IFN-γ) ELISPOT, intracellular cytokine staining (ICS) followed by flow cytometry (FACS) and by RT-qPCR for expression of IFNγ responsive genes; monokine induced by IFNγ (MIG, CXCL9) and interferon inducible protein 10kDa (IP-10, CXCL10). ICS will be used to assess antigen specific polyfunctional cells that make IFNγ, tumour necrosis factor-alpha (TNFα), inetrleukin-2 (IL-2) and macrophage inflammatory protein-1β (MIP-1β). Staining for perforin, granzyme B and CD107a (LAMP-1) will be used to assess cytotoxic function. Standard operating protocols will be developed and used for each assay.

Concurrently serum samples will be used to measure the appearance of specific neutralising antibody to JEV SA14-14-2 and to a representative South Indian wild type virus, JEV P20778 (Vellore strain).

Blood will be stored in PAXGENE at -80°C for subsequent RNA extraction. RNA will be extracted from participants' blood pre-vaccination, at day 7 and 8 weeks post vaccination. A whole blood transcriptomic approach will be used to investigate changes in gene transcription following vaccination.

Responses will be tracked over time and subsequent samples will be used for fine mapping of peptides to determine the minimal epitopes for CD4 and CD8 T cells. Tetramers of HLA class I and minimal peptides will be made and used to investigate the changes in antigen specific cells over time as well as their expression of activation markers, chemokine receptors, and other functional molecules. These experiments will be used to compile a complete picture of the breadth, magnitude, quality and duration of the T cell response to live vaccine JEV SA14-14-2.

7. ETHICAL CONSIDERATIONS

In accordance with this clinical trial protocol and applicable laws and regulations including, but not limited to, the International Conference on Harmonisation Guideline for Good Clinical Practice (GCP) and regulatory requirements of the Indian Drug Controller and local Indian rules and regulations. The institutional human ethics committee (IHEC) of IISc will review and approve the protocol and informed consent form (ICF) before any participants are enrolled. The participant will be consented using the approved ICF before any data specified in the protocol are collected.

7.1 Consent

All participants will only be recruited if they freely give their fully informed consent to participate in the study. There will be no external pressure or inducement for participation. Participants will be free to withdraw at any point in the study for any reason or without giving a reason. This will be made clear in the consenting process. Participants will also be entitled to ask for the destruction of any stored samples at any point in the study. It should be noted that the planned experiments by their nature result in the destruction of the samples by default. Any person thought not to understand the consent process or to not be capable of giving consent will not be recruited. In the event of a participant losing capacity for consent durint the study they will be withdrawn from the study and any unused samples destroyed. However any data generated will still be used.

7.2 Risks

SA14-14-2 is licensed for use in India where millions of doses have been delivered since 2006. At the beginning of the campaign in 2006 there were 9.3 million doses of SA14-14-2 JE vaccine delivered with 65 serious adverse events and 22 deaths reported; however upon investigation there was no clear link with SA14-14-2 vaccination. In recent years three rigorously conducted studies have recorded no serious adverse events. Although the SA14-14-2 JE vaccine is not yet pre-qualified by the World Health Organisation, work towards this is ongoing. However, the vaccine has been reviewed by the Global Advisory Committee on Vaccine Safety (GACVS) of the WHO and was found to have a good safety profile. The very wide experience of use of SA14-14-2 JE vaccine in many countries in Asia makes the occurrence of any serious risk very unlikely.

7.3 Benefits

participants will be screened for immunity against JEV and WNV. Participants who are not immune to JEV will be vaccinated and hence will become immune to the disease. There is the possiblity of some immunity against DENV, based on experience from previous JE vaccination studies.

7.3.1 Advantage of live JE vaccine

The cell culture-based, live attenuated vaccine SA 14-14-2 appears to require fewer doses for long term protection, is in most cases less expensive, and seems to represent an attractive alternative to the mouse-brain derived vaccine. Mouse-brain vaccine gives a limited duration of protection, the need for multiple doses, and a high price per dose. Mouse-brain derived vaccine likely gives more adverse reactions than live attenuated vaccine SA 14-14-2.

7.3.2 Duration of immunity

In general, live vaccines elicit stronger cell mediated immune responses. This in turn may lead to a longer degree of immune protection than the killed vaccine. For example live yellow fever vaccine gives detectable neutralising antibodies up to 35-40 years after a single dose. Childhood protection against JE is obtained by a single dose of the cell-culture based, live attenuated SA 14-14-2 vaccine followed by a single booster given at an interval of about 1 year.

7.3.3 Theoretical basis of cross protection with DENV

A placebo controlled study of mouse brain derived inactivated JE vaccine showed a small reduction in dengue disease in people who received JE vaccine though, this did not quite reach statistical significance. Whether there is any protective effect of live JE vaccine on dengue disease is unknown, but based on previous data, there may be a small beneficial effect.

7.3.4 Important need for knowledge on T cell function

As a general remark, it is noteworthy that T cell effector function helps to prevent disease, but does not necessarily prevent infection, as exemplified by cytotoxic T Lymphocytes (CTL) that attack and kill cells, once they have become infected with virus. This mechanism may help to limit virus production and dissemination in the tissue and act as an adjunct to neutralising antibody. Moreover, T cells help to produce longer lasting and higher titre antibody responses and efficient anamnestic immune responses.

In general the field of vaccine research is hampered by the difficulty is showing clinically significant immune protection due to the large sample sizes needed. Frequently surrogates of protection such as neutralising antibody titre are used. Despite abundant data showing

that T cells respond to vaccination, there are few T cell assays validated as endpoints in clinical studies. More knowledge of T cell responses across different vaccines is needed to inform such endpoints and broaden the basis of knowledge for long-lived and robust immunity.

Anti-Flavivirus immune responses are significantly cross-reactive without necessarily conferring protection and sometimes even disease is enhanced (e.g. dengue shock syndrome). In order to better understand immune correlates of protection, identify potentially confounding cross-reacting epitopes that might confuse future vaccine studies (e.g. dengue vaccines), and investigate the potential for cross-priming by other flaviviruses a detailed description of cellular immune outcomes of JE vaccination is desirable. An understanding of the normal T cell immune response to JEV may also be inferred from this study, regarding vaccination as a model of viral infection where no disease results. These data may inform subsequent studies into the possible role of T cells in the pathogenesis of acute JE.

7.4 HIV testing

All participants in the study will need to be HIV tested because HIV infection as the potential to profoundly affect immune responses. Also, little is known about the safety of SA14-14-2 in HIV+ individuals. Although the vaccine, must have been given to HIV+ individuals many times in the past unknowingly, this has always been in the context of large public health campaigns in areas of low to moderate HIV endemicity where the potential benefit of vaccination to such a potentially devastating disease outweighs the risks in public health terms. This would not be the case in phase 1 of this study where the risk would not be justified outside the setting of a study specifically designed to address this question, which this study is not. Rare but serious adverse reactions have been reported following the use of yellow fever 17D live attenuated vaccine in a subject with unknown advanced HIV disease [36]. For these reasons HIV+ individuals must be excluded from this study. HIV testing of children is always contentious because it is in effect to HIV test the parents as well. This will be explained at the time of study entry. HIV testing of children on similar studies to this has been conducted in Karnataka state before. Anti-retroviral therapy is increasing widely available in India and is available in Bangalore (for example there is an HIV clinic at NIMHANS). Opportunistic infection prophylaxis is also available and can also improve the prognosis independently of antiretroviral therapy. Knowledge of HIV status can also modify behaviour and limit transmission. Therefore, overall, there would be a benefit to all participants (adults, children and parents) learning their previously unknown HIV status. Any participant found to be HIV+ will be counselled by the study team and referred to local health services for ongoing care.

8. QUALITY CONTROL AND QUALITY ASSURANCE

The sponsor implements and maintains quality assurance and quality control systems with written SOPs to assure that trials are conducted and data are generated, recorded, and reported in compliance with the protocol, GCP and the Indian national regulatory requirement for investigational product. A trial initiation visit will be done prior to the enrollment of any volunteer at a site. The clinical monitor and investigator will review the protocol, logistics and all trial related procedures. This includes information on the vaccine, procedures for obtaining informed consent, procedures for reporting SAEs and procedures for completing the CRFs. Site monitoring visits will be scheduled by the clinical monitor on a regular basis. During these visits, information recorded in the CRFs will be verified against source documents for accuracy and completion. The clinical monitor will review the informed consent procedure, product accountability and storage, trial documents and trial progress. The clinical monitor will verify that the investigator follows the approved protocol or amendments (if any). He/she will observe trial procedures and will discuss any problems with the investigator. Monitoring visits will be recorded in the Monitoring Log at the investigator's site, and at the end of the trial a copy of the completed log will be returned to the sponsor.

Personal volunteer data will be kept confidential. The file cabinets with the trial data and the volunteer information will be locked and accessed only by authorized persons from the sponsor and Regulatory Authorities. CRFs or other documents submitted to the sponsor will identify a volunteer by the participant's initials and study enrollment number only. The investigator will keep in the investigator's files a participants Identification List + Screening/enrolment Log (including complete name, age and address). To allow compliance with GCP principles, each volunteer will be asked for consent regarding direct access to the source documents for monitoring, audit, and inspections. The agreement covering the use of the data or analysis has to be documented in writing, together with the written informed consent for trial participation.

9. CONFIDENTIALITY

All study related documents will be kept in locked cabinets at the study site. Participant;s names will appear on the screening documents and once enrolled study documents will refer to the participant's initials and assigned study code number. Only personnel involved with the study conduct and local and international regulatory agencies may review these records. The privacy of all participants will be protected in so far as permitted by law.

9.1 Direct access to source data/documents

The investigators will provide written agreement that the investigator(s) /institution(s) will permit trial-related monitoring, audits, IRB/IEC review, regulatory inspection(s) and providing direct access to source data/documents, to sponsors, study monitors and the relevant authorities.

9.2 Data handling and Record Keeping

All trial data will be recorded on the CRFs. Only the investigator and authorized co-workers, according to the list of Authorized Signatory Form (ASF), are authorized to make entries on the CRF. The CRFs would be completed in English.

Source documents including medical records and original laboratory results will be kept in a separate file at the investigator's office. CRFs will be kept in a room with limited access.

After completion of the study the investigators will maintain all study documentation for three years.

10. FINANCING AND INSURANCE

This study will be funded by Wellcome trust of Great Britian. The sponsor of the trial will be The Indian Institute of Science. This study will not cover any health insurance coverage as this a phase IV study of a licensed vaccine already being administered in India.

11. PUBLICATION POLICY

All the results from this study would be published in appropriate international scientific journal and due credits would be shared on the basis of contribution from all those involved in this vaccine trial. The data would be shared as required by the regulators.

All rights and interests worldwide in any inventions, know-how, or other intellectual or industrial property rights which arise during the course of and/or as a result of the clinical study which is the participants of this protocol or which otherwise arise from the information or materials supplied under this Protocol, shall be assigned to, vest in and remain the property of the Indian Institute of Science.
